# EZH2 activates CHK1 signaling to promote ovarian cancer chemoresistance by maintaining the properties of cancer stem cells

**DOI:** 10.7150/thno.48101

**Published:** 2021-01-01

**Authors:** Yiping Wen, Yaya Hou, Xiaoqing Yi, Si Sun, Jing Guo, Xiaoqi He, Tao Li, Jing Cai, Zehua Wang

**Affiliations:** Department of Obstetrics and Gynecology, Union Hospital, Tongji Medical College, Huazhong University of Science and Technology, Wuhan 430022, China.

**Keywords:** Cancer stem cell, chemoresistance, ovarian cancer, EZH2 and CHK1

## Abstract

**Background:** Ovarian cancer is a fatal malignant gynecological tumor. Ovarian cancer stem cells (OCSCs) contribute to resistance to chemotherapy. The polycomb group protein enhancer of zeste homolog 2 (EZH2) plays a key role in maintaining CSCs. Here, we aimed to investigate the specific mechanism by which EZH2 regulates CSCs to result in chemoresistance and poor prognosis of ovarian cancer.

**Methods:** We used a nude mouse model to obtain a cell line enriched for OCSCs, named SK-3rd cells. The CRISPR and Cas9 endonuclease system was used to establish an EZH2-knockout SK-3rd ovarian cancer cell line. High-throughput PCR array and bioinformatics methods were used to screen the EZH2 target involved in CSC stemness. A luciferase reporter assay and chromatin immunoprecipitation assay were performed to identify activation of CHK1 by EZH2. We evaluated associations between EZH2/CHK1 expression and the chemoresistance and prognosis of ovarian cancer patients.

**Results:** EZH2 plays a critical role in maintaining ovarian CSC stemness and chemo-resistance. CHK1 is an EZH2 target involved in CSC stemness. Knockdown of EZH2 in ovarian CSCs decreased CHK1 expression, while CHK1 overexpression was sufficient to reverse the inhibitory effect on spheroid formation and chemoresistance caused by repression of EZH2. In addition, EZH2 was also shown to play a unique role in activating rather than repressing CHK1 signaling through binding to the CHK1 promoter in epithelial ovarian cancer cells. Finally, in clinical samples, ovarian cancer patients with high levels of EZH2 and CHK1 not only were more resistant to platinum but also had a poorer prognosis.

**Conclusions:** Our data revealed a previously unidentified functional and mechanistic link between EZH2 levels, CHK1 signaling activation, and ovarian CSCs and provided strong evidence that EZH2 promotes ovarian cancer chemoresistance and recurrence.

## Introduction

Epithelial ovarian cancer (EOC) is one of the most lethal tumors among gynecological malignancies. Due to a highly invasive growth pattern and frequent resistance to chemotherapy, the prognosis for patients with EOC is poor 1. Previous reports have indicated that cancer stem cells (CSCs), which are characterized by self-renewal capacity and tumorigenicity, are associated with the chemo-resistance, progression and recurrence of EOC 2-4. Therefore, understanding the molecular mechanisms that sustain the stemness of tumor cells will provide new strategies to improve ovarian cancer treatments.

Enhancer of zeste homolog 2 (EZH2), a member of the polycomb repressive complex 2 (PRC2), is known to catalyze trimethylation of lysine 27 on histone 3 (H3K27me3) and consequently induce transcriptional repression of target genes. It has been reported that EZH2 is upregulated in multiple malignancies, such as breast cancer, prostate cancer, melanoma and ovarian cancer 5-9, where its oncogenic activity has been proven to be primarily mediated by silencing tumor suppressor genes 6, 10. Recent evidence implicates EZH2 in transcriptional activation 11-15, but the mechanisms are not well defined. A previous report identified that EZH2 overexpression is significantly associated with chemoresistance and independently predicts a poor prognosis for EOC patients 16. In addition, EZH2 has been shown to be upregulated in the side population of ovarian cancer cells, which are stem cell-like cells that are enriched by chemotherapy, suggesting that overexpression of EZH2 in ovarian CSCs may contribute to chemoresistance 17. Additionally, EZH2-specific microRNA-98 could effectively inhibit cell proliferation *in vitro* and regulate the pRb-E2F pathway in human epithelial ovarian cancer stem cells (EOCSCs) 18. Another study showed that the expression of EZH2 was positively correlated with c-KIT (CD117), a surface marker of EOCSCs, and that EZH2 inhibition may represent an effective therapeutic strategy against ovarian cancer 19. Collectively, these findings link EZH2 to EOCSCs. However, the underlying mechanism regarding the effects of EZH2 on EOCSC stemness maintenance remains elusive.

In the present study, we uncovered a novel molecular mechanism by which EZH2 confers EOCSC stemness and chemoresistance. Inhibition of EOCSCs through EZH2 repression represents a promising therapeutic strategy in EOC treatment. Thus, we highlight a new aspect of EOC biology and provide a molecular basis for further exploration of EZH2-mediated anti-EOC targeted therapy.

## Materials and Methods

### Primary tumor specimens

This study was conducted according to the principles expressed in the Declaration of Helsinki and was approved by the Research Ethics Committee of Union Hospital, Tongji Medical College, Huazhong University of Science and Technology (Wuhan, China). Written informed consent was obtained from 12 chemotherapy-naive patients and 4 ovarian cancer patients who received 2-3 courses of platinum-based neoadjuvant chemotherapy. All 16 patients underwent 6 courses of platinum-based adjuvant chemotherapy after surgery. The chemotherapy regimen in both the neoadjuvant and adjuvant settings was a combination of paclitaxel (135-175 mg/m^2^) and carboplatin (dosage according to a creatinine clearance value of 5 or 6) (**Table S1**). In clinical practice, patients in whom complete clinical remission is not achieved after initial platinum-based therapy or tumor relapse occurs within 6 months after complete remission are defined as platinum-resistant cases, while those with a platinum-free interval longer than 6 months are defined as platinum-sensitive cases 20. The procedures were carried out in accordance with the university's scientific research guidelines and regulations. All samples were received in the laboratory within 30 min and immediately mechanically disaggregated and digested with collagenase. Single cell suspensions were obtained by filtration through a 40 µm filter.

### Cell lines

Human EOC cell lines SKOV3 (adenocarcinoma), A2780 (adenocarcinoma), IGROV1 (endometrioid adenocarcinoma) and OVCAR4 (high-grade serous adenocarcinoma) 21 were obtained from the China Center for Type Culture Collection (CCTCC, Wuhan University, China) and grown under the recommended conditions. All of the cell lines we used in the experiments were confirmed to be free of mycoplasma using the Mycoplasma Detection Kit (CA1080, Solarbio, Beijing, China) and authenticated by short tandem repeat profiling (Shanghai Biowing Applied Biotechnology Co. LTD).

### Cell culture and spheroid culture

All adherent cells were maintained in DMEM/F12 medium supplemented with 10% (v/v) fetal bovine serum at 37 °C in a humidified atmosphere of 5% CO2. Spheroids were generated from cells after plating at a density of 1000 cells/well in ultra-low attachment 6-well culture plates (Corning, NY, USA). Spontaneously generated spheroids were cultured in serum-free DMEM/F12 medium supplemented with 2% B-27 Supplement (Invitrogen, Carlsbad, CA, USA) without vitamin A, 20 ng/mL basic fibroblast growth factor (FGF, Peprotech, Rocky Hill, NJ, USA), 20 ng/mL epidermal growth factor (EGF, Peprotech), 10 ng/mL leukemia inhibitory factor (LIF, Peprotech) and insulin-transferrinselenium (ITS, Invitrogen). Fresh medium was added every 3 days, and spheroids were cultured for approximately 2 weeks before they reached a diameter of approximately 150 μm. The spheres were collected by gentle centrifugation, dissociated with accutase (Invitrogen) and mechanically disrupted with a pipette. The resulting single-cell suspension was then centrifuged and resuspended in serum-free medium to allow reforming of spheres.

### Transfection *in vitro*

GFP-labeled lentivirus encoding EZH2 shRNA (shEZH2) and a nontargeting shRNA (shNC) were purchased from GenePharma (Suzhou, China). The sequence of EZH2-shRNA was 5′-GCAACACCCAACACTTATAAG-3′, and the negative-control shRNA sequence was 5′-TTCTCCGAACGTGTCACGT-3′. Stable transfection of EZH2 knockdown lentivirus was conducted following manufacturer's instructions. Approximately 48 h after transduction, the medium was changed, and puromycin (2 μg/mL) was added for selection of stably transduced cells until all cells were GFP positive, as detected by microscopy.

The siRNAs targeting *CHK1*, *EED*, and *SUZ12* as well as control siRNA were obtained from GenePharma. The siRNA targeting sequences are shown in **Table S2**. Transient transfection of siRNA was conducted using Lipofectamine 2000 transfection reagent (Invitrogen) according to the manufacturer's instructions. After transfection for 48 h, cells were harvested for further analysis.

Constructs containing wild-type EZH2 and EZH2 ∆SET were generated by the insertion of cDNA encoding full-length EZH2 and its SET domain-deficient mutant (ΔSET) into CMV-MCS-3FLAG-SV40-Neomycin vectors (Genechem, Shanghai, China). The EZH2-overexpressing plasmid (CV061/CMV-MCS-3FLAG-SV40-Puromycin) and CHK1-overexpressing plasmid (pcDNA3.1 (-)/CMV-MCS-SV40-Neomycin) were purchased from Genechem. Cells were transfected with plasmid DNA and negative control using Lipofectamine 3000 transfection reagent (Invitrogen) according to the manufacturer's protocol. After transfection for 48 h, cells were harvested for further analysis.

### Establishment of gene-knockout cell lines using the CRISPR/Cas9 system (CRISPR and Cas9 endonuclease system)

Cells were generated using a CRISPR-Cas9 strategy. The Cas9 lentivirus and lentiviral sgRNA vectors targeting EZH2 (sgEZH2) or scrambled control (sgNC) was purchased from Genechem (Shanghai, China). The sequence for the sgRNA targeting EZH2 was 5'-TGCGACTGAGACAGCTCAAG-3', and the negative-control sgRNA sequence was 5'-TTCTCCGAACGTGTCACGT-3', Cas9 lentiviral particles harboring a puromycin-resistance marker were transfected into SK-3rd cells. Lentiviral particles were added to cells at a multiplicity of infection of 10. After 24 h, the medium was replaced with full medium and cells were then passaged into selective medium with 2 μg/mL of puromycin until resistant Cas9-expressing cell (SK-3rd-cas9) colonies formed and antibiotic-induced cell death ceased. Then, the SK-3rd-cas9 cells were transfected with lentiviral vectors expressing both GFP and sgRNA targeting EZH2 (SK-3rd/sgEZH2). A scrambled control vector served as the negative control (SK-3rd/sgNC). After transfection, cells were incubated for 72 h. GFP-positive cells were isolated using limiting dilution and formed a single colony. The knockout efficiency was confirmed by Western blotting.

### Animal studies

The animal experiment was supervised and approved by the Institutional Animal Care and Use Committee of Tongji Medical College, Huazhong University of Science and Technology.

To acquire the chemoresistant SK-3rd cell lines, human EOC cell lines SKOV3 (6.0 × 10^6^ cells/0.2 mL in phosphate buffered saline) was passaged in 4-week-old female BALB/c-nu nude mice (Beijing Vital River, China) by subcutaneous injection into the right side of the interscapular region. When the solid tumor xenograft reached an approximate diameter of 0.5 cm, cisplatin (3 mg/kg/d, Qilu pharmaceutical company, China), dissolved in 0.9% sterile saline, was injected into the tumor every other day. An equal volume of 0.9% sterile saline was injected into the tumors of the control mice. Single-cell suspensions of tumor xenografts, which were removed when tumors reached ~2 cm in diameter, were obtained by collagenase digestion. Dissociated cells treated with cisplatin were cultured in suspension and the control cells were cultured in adherence. Approximately two weeks later, the two groups of cells were repetitively passaged in cisplatin-treated or saline-treated female BALB/c-nu nude mice, respectively, as above. Freshly isolated tumor cells obtained from the first-, second-, and third-generation xenografts treated with cisplatin were named SK-1st, SK-2nd, and SK-3rd cells, respectively, and the control cells obtained from xenografts treated with 0.9% sterile saline were named SKOV3 cells.

For tumorigenicity experiments, 4-week-old female BALB/c-nu mice were randomly divided into two groups (six mice in each group). The SKOV3 and SK-3rd cells, or SK-3rd/shNC and SK-3rd/shEZH2 cells, were diluted (10^4^ and 10^5^ cells) and mixed with Matrigel (BD Biosciences, San Jose, CA, USA) at 1:1. The mixtures were implanted subcutaneously into the right side of the interscapular region of mice. On the indicated dates after cell injection, tumor volumes were determined using an external caliper. The following formula was used to calculate tumor volume: tumor volume (mm^3^) = (L × W^2^) × 0.5 (L, length; W, width). Mice were sacrificed by cervical dislocation, and the presence of tumors was confirmed by necropsy.

### PCR microarrays

RT^2^ Profiler PCR Arrays Human Cancer Stem cells (catalog no. PAHS-176Z) from SA Biosciences were used to identify the gene expression profiles of SK-3rd/shNC and SK-3rd/shEZH2 cells. The arrays were performed in triplicate for each condition.

### Flow cytometry

Flow cytometry was used to detect stem cell markers (ALDH1, CD44, CD24, and CD133) and lineage (Lin) markers (CD235a, CD45, CD31 and CD140a) in ovarian cancer cells. The ALDEFLUOR Kit (STEMCELL Technologies, Vancouver, Canada; catalog no. 01700) was used to determine the levels of ALDH1 in ovarian cancer cells following the manufacturer's instructions. For detection of other stem cell markers and Lin markers, 1 × 10^6^ cells were labeled with antibodies at the manufacturer's recommended dilutions (**Table S3**) and analyzed or sorted on a Moflo Beckman Coulter (Fullerton, CA, USA) or a LSRII Fortessa (BD Biosciences, CA, USA).

### Bioinformatics analysis

Data for EZH2 and CHK1 expression in primary ovarian cancer tissues were downloaded from The Cancer Genome Atlas (TCGA) (https://tcga-data.nci.nih.gov/tcga/), and three different Affymetrix microarray datasets-GSE9891 (285 serous and endometrioid tumors of the ovary, peritoneum, and fallopian tube) 22, GSE14764 (80 frozen ovarian cancer samples) 23 and GSE26712 (185 previously untreated late-stage (III-IV) high-grade ovarian cancer samples and 10 normal ovarian surface epithelium samples) 24-were downloaded from the Gene Expression Omnibus website (GEO, http://www.ncbi.nlm.nih.gov/geo/). STRING 10, a search tool for the retrieval of interacting genes/proteins (http://string-db.org/), was used to construct the protein-protein interaction (PPI) between proteins encoded by differentially expressed genes. The prognostic significance of EZH2 and CHK1 in ovarian cancer patients was analyzed by data mining in the KM-Plotter plotter database (http://kmplot.com). Kaplan-Meier survival curves and log-rank statistics were used to evaluate the difference in progression-free survival (PFS) time and overall survival (OS) time. Pearson correlations were used to estimate bivariate correlations between EZH2 and CHK1 family genes.

### Inhibitors

AZD7762 (CHK1 inhibitor), AZD6738 (ATR inhibitor), EPZ6438 (EZH2 inhibitor, also called Tazemetostat), KU-60019 (ATM inhibitor) were purchased from Selleckchem (Houston, TX, USA). GSK126 (an inhibitor of EZH2 methyltransferase activity) was purchased from Cayman Chemical (Ann Arbor, Michigan, USA). All drugs were dissolved in dimethyl sulfoxide (DMSO, Sigma-Aldrich, St. Louis, MO, USA) and stored at -20 °C.

### Cell cycle distribution

The cell cycle distribution was analyzed by flow cytometry. After treatment with cisplatin combined with the abovementioned inhibitors or DMSO for 48 h, EOC cells were harvested and fixed in 75% ice-cold ethanol for approximately 24 h at 4 °C. Subsequently, the cells were washed gently with cold PBS twice, incubated with propidium iodide (50 μg/mL) in a buffer containing 50 μg/mL Rnase and then stored at 4 °C overnight before analysis. The cell cycle distribution was assessed by FACScan (BD Biosciences), and the results were analyzed with Modfit LT 3.2 software (Verity Software House, Topsham, ME, USA).

### Apoptosis analysis

Apoptosis in SKOV3 or SK-3rd cells cultured in the presence or absence of cisplatin (Qilu pharmaceutical company, China) at a concentration of 20 μM for 48 h was detected using the Annexin-V-FITC/PI Apoptosis Detection Kit (BD Biosciences, San Jose, CA, USA) according to the manufacturer's instructions. Briefly, cells were washed twice with cold PBS. After setting aside the blank group of cells as a negative control, the other groups were resuspended in 100 μL of Annexin V binding buffer (0.1 M HEPES, M NaCl, 2.5 mM CaCl_2_), and then incubated with 5 µL FITC Annexin V and 5 µL PI for 15 min at room temperature in the dark. Then, the cells were examined using a MoFlo XDP cell sorter (Beckman Coulter, Brea, CA, USA).

To determine the functional roles of EZH2 and CHK1 in apoptosis, GFP-tagged Sk-3rd/sgNC and SK-3rd/sgEZH2 cells with or without CHK1 over-expression were exposed to cisplatin at a concentration of 20 μM for 48 h. Then, apoptotic cells were quantified with an Annexin V-PE/7-AAD apoptosis detection kit (BD Biosciences). Cells were washed twice with cold PBS followed by resuspension in 100 μL binding buffer at a concentration of 1 × 10^6^ cells/mL. Cells were incubated with 5 μL 7-AAD and 5 μL Annexin V-PE for 15 min. Detection was conducted within 1 h using a MoFlo XDP cell sorter (Beckman Coulter, Brea, CA, USA). The number of early apoptotic cells, late apoptotic cells or necrotic cells was determined by counting the percentage of Annexin V-PE+/7-AAD- cells, Annexin V-PE+/7-AAD+ cells or Annexin V-PE-/7-AAD+ cells, respectively (“+” indicates positive staining; “-” indicates negative staining). Annexin V-PE-/7-AAD- cells were considered surviving cells.

### Neutral comet assay

The neutral comet assay was performed using Comet assay kits (Trevigen, Maryland, USA) according to the manufacturer's instructions. SYBR green I nucleic acid stain (Biosharp, Wuhan, China) was used to stain DNA. Images were taken on an epifluorescence microscope (Olympus IX71, Olympus, Japan) at × 10 magnification. Tail moments were analyzed using OpenComet software (www.cometbi.org/) 25.

### Colony formation assay

Ovarian cancer cells were seeded in six-well plates at a density of 500 cells per well and cultured until visible colonies appeared. The colonies were fixed with 4% formaldehyde for 30 min and stained with 0.1% crystal violet for at least 1 h, and those consisting of more than 50 cells per colony were counted manually. All assays were performed at least in three independent experiments.

### Immunofluorescence

After treatment with cisplatin, cells were assessed via immunofluorescence for unrepaired DNA damage via phosphorylation of H2AX (pH2AX), a standard marker of unrepaired double-strand DNA damage. For these experiments, cells were grown on coverslips. All groups of cells were fixed in 4% paraformaldehyde with 0.1% Triton-X100 and probed with an anti-pH2AX primary antibody (**Table S3**) overnight at 4 °C followed by an anti-rabbit-Cy3 secondary antibody (**Table S3**) for 1 h at room temperature. After PBS washing, the cells were counterstained with 4′, 6-diamidino-2-phenylindole (DAPI) to mark the nuclei. The coverslips were placed onto slides, and the foci were visualized under an A1-Si Nikon confocal laser scanning microscope (Nikon, Tokyo, Japan).

### MTT assays

MTT assays were performed to evaluate the cytotoxicity of the treatments. Briefly, cells were seeded at 5, 000 cells/well in 96-well plates (Corning, NY, USA) in 100 μL DMEM/F12 medium supplemented with 2% fetal bovine serum (FBS) for 24 h at 37 °C. The cells were then pretreated with 0 to 100 μM cisplatin for 48 h (N = 3 per drug dose), followed by treatment with 100 μL sterile MTT (3-(4, 5-dimethylthiazol-2-yl) 2, 5-diphenyl tetrazolium bromide) dye (0.5 mg/mL, Sigma, St. Louis, MO, USA) for 4 h at 37 °C. Following incubation, 100 μL of extraction buffer was added to each well. After overnight incubation, the absorbance was determined at 570 nm on an iMark microplate reader (Bio-Rad, Hercules, California, USA; Serial No. 10601). The 50% inhibitory concentration (IC50) values were calculated using linear interpolation.

### Western blot analysis

Details about the antibodies used are shown in**Table S3**. Cells were washed with ice-cold PBS and then directly lysed in cold radioimmunoprecipitation assay (RIPA) buffer (Beyotime Biotechnology, Shanghai, China), including a protease inhibitor cocktail, for 30 min. Cellular lysates were then clarified by centrifugation at 13,000 g for 10 min to collect supernatants. The protein concentration was measured with a bicinchoninic acid (BCA) assay, and then samples were separated by 10% sodium dodecyl sulfate-PAGE (SDS-PAGE) and transferred onto a PVDF membrane. The membranes were then blocked with 5% nonfat milk in Tris-buffered saline with Tween-20 (TBST) for 1 h at room temperature and incubated with primary antibody at 4 °C overnight. Horseradish peroxidase-conjugated anti-rabbit or anti-mouse secondary antibodies were subsequently used. Protein bands were detected with an enhanced chemiluminescence kit (Pierce, Thermo Scientific, Waltham, MA, USA) in Molecular Imager® ChemiDoc^TM^ XRS+ with Image Lab^TM^ Software (Bio-Rad Laboratories, Hercules, CA, USA).

### Transcript level analysis

Total RNA was extracted using TRIzol Reagent (Invitrogen) for reverse transcription-PCR (RT-PCR) and real-time quantitative PCR (qRT-PCR) analysis. qRT-PCR analysis was performed using SYBR Green (Takara Bio Inc., Japan). The primers used for transcriptional analysis are listed in **Table S4**.

### Luciferase reporter assay

SKOV3 and A2780 cells were seeded in 24-well plates (1.5 × 10^3^ cells/well) in triplicate. After 24 h of culture, the cells were cotransfected with the desired firefly luciferase reporter plasmid (400 ng/mL, Genepharma) and TK-Renilla luciferase plasmid (20 ng/mL, Genepharma) using Lipofectamine 2000 reagent. Luciferase and Renilla signals were determined 24 h after transfection using a Dual Luciferase Reporter Assay Kit (GN201-01, YPH-bio, Co. Ltd. Beijing, China) on a GloMax 20/20 luminometer (Promega, Madison, WI, USA).

### Chromatin immunoprecipitation assay (ChIP)

The putative promoter regions (0.5 kb upstream and 2 kb downstream of the transcription start site) of the *CHK1* gene were retrieved from the UCSC Genome Browser (http://genome.ucsc.edu/). The ChIP assay was performed according to the manufacturer's instructions for the EpiQuik Chromatin Immunoprecipitation (ChIP) Kit (Epigentek, Farmingdale, NY, USA). Cells (2×10^6^) plated in a 100-mm culture dish were treated with 1% formaldehyde to cross-link proteins to DNA. The cell lysates were sonicated to shear the DNA into 300-1000-bp lengths. Aliquots containing equal amounts of chromatin supernatants were incubated on a rocking bed at 4 °C overnight with either 1 μg anti-EZH2 antibody, 1 μg anti-H3 antibody, or 1 μg anti-IgG antibody as a negative control. Following reverse cross-linking of protein-DNA complexes to free the DNA, qRT-PCR was carried out. Human *HOXA2* promoter primers were used as positive controls, and α-satellite repeat primers (Cell Signaling #4486) were used as negative controls. The primers used in this study are listed in**Table S5**.

### Immunohistochemistry

The EZH2 and pCHK1 (Ser345) expression levels were examined in 44 paraffin-embedded EOC tissues. The clinical and pathological characteristics of the patients from whom the tissues were collected are summarized in **Table S6.** Paraffin-embedded xenograft samples were used for the immunohistochemical staining of EZH2, Ki-67, ALDH1, E-CADHERIN and VIMENTIN. Endogenous peroxidase activity was blocked by treatment with 3% hydrogen peroxide in PBS for 30 min. The specimens were rinsed in PBS. The sections were incubated with primary antibodies (**Table S3**) overnight at 4 °C. The bound antibody was detected with a secondary biotinylated antibody for 30 min at room temperature and visualized using diaminobenzidine as a chromogenic substrate. The sections were then counterstained with hematoxylin. Positive expression was defined as brown-yellow granules in the cytoplasm or nucleus. The stained sections were evaluated and scored by two independent pathologists blinded to clinical and pathological data, based on both the percentage of positive cells (0, <10%; 1, 10%-20%; 2, 21%-50%; and 3, >50%) and the staining intensity (0, negative; 1, faint; 2, moderate; and 3, strong). Their product formula represents the final integral of each sample. Only cells associated with tumor islets were scored, and stromal and vascular staining was not evaluated. An immunohistochemistry (IHC) score less than 3 indicated low expression of EZH2 or pCHK1, while a score of 3 or greater indicated high expression. The controls used were isotype-matched IgGs.

### Statistical analysis

All data were expressed as the mean±SD (N ≥ 3). Student's t test or one-way analysis of variance was used to evaluate differences between two or multiple groups, respectively. Nonparametric data were analyzed using the Kruskal-Wallis test. Pearson's correlation coefficient was used to identify the correlation between EZH2 and pCHK1 in EOC tissues. Kaplan-Meier survival curves and log-rank tests were used to present survival differences between groups. A multivariate analysis of risk factors for PFS was performed using a Cox proportional hazards model. A logistic regression analysis was carried out to assess the independent factors that may have an impact on the platinum resistance of patients. A value of P < 0.05 was considered statistically significant. Data analyses were carried out using the SPSS 16.0 statistical software package (IBM, Armonk, NY, USA).

## Results

### CSC enrichment after chemotherapy

**Figure 1:**
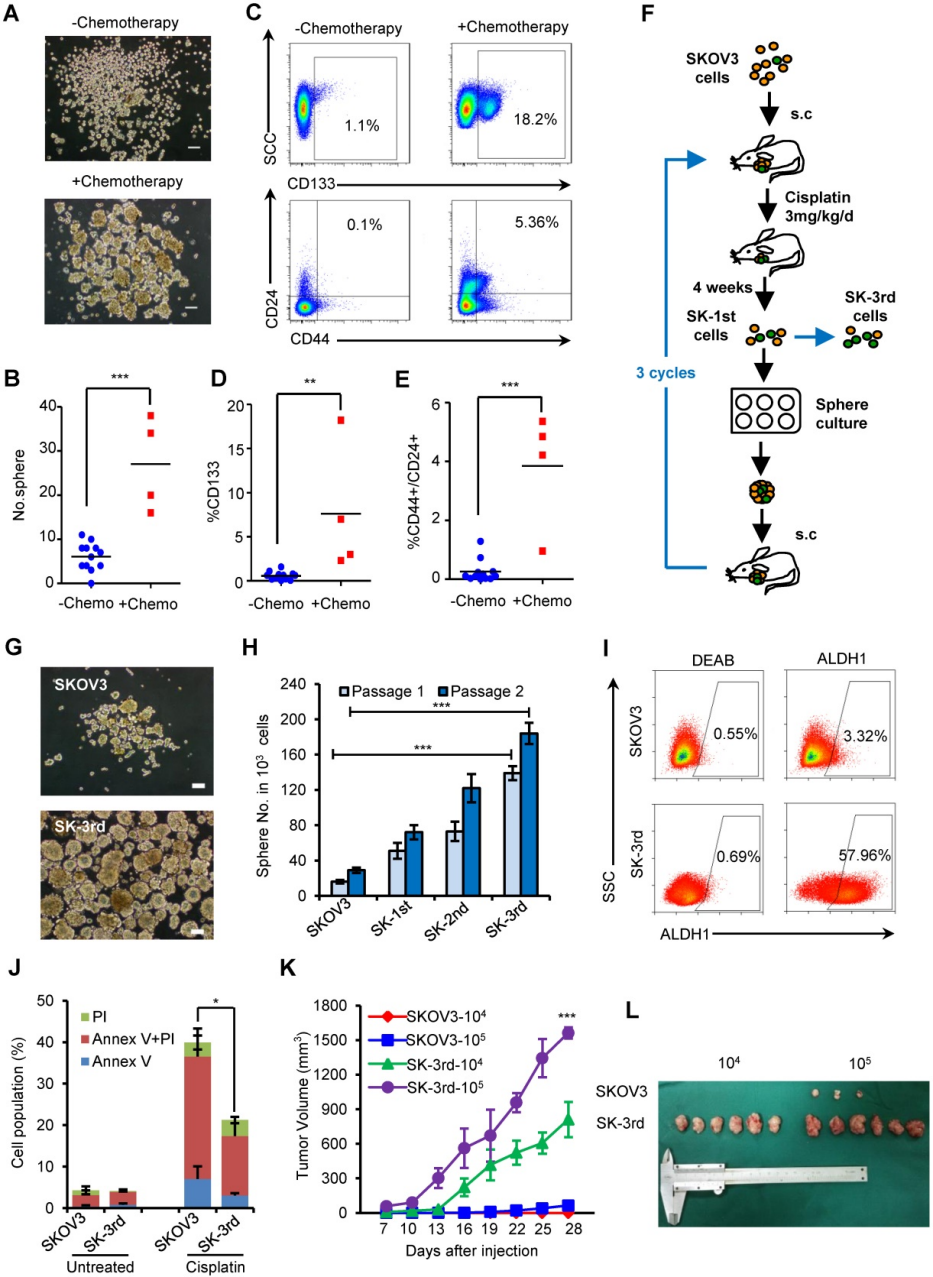
** Chemotherapy selectively enriches for self-renewing ovarian CSCs. (A-C)** Ovarian cancers from 4 patients who received neoadjuvant chemotherapy were substantially enriched for self-renewing cells with the expected properties of ovarian CSCs compared to those from the 12 untreated patients. Representative images showing increased numbers of spheres after 14 days of culture **(A, B)** and increased percentages of CD44+/CD24+/Lin- and CD133+/Lin- cells in freshly isolated tumors from a patient who received chemotherapy **(C)**; Scale bar: 50 µm. **(D, E)** Flow cytometric assays detected a higher percentage of CD133+/Lin- (D) and CD44+/CD24+/Lin- (E) populations in cells from patients who received neoadjuvant chemotherapy than from chemotherapy-naive patients. **(F)** Schematic showing the xenotransplantation approach, as described in the Materials and Methods. **(G, H)** Representative images **(G)** and a statistical histogram **(H)** showing the increased numbers of spheres formed by SK-3rd cells compared with the SKOV3 cells on day 14 from 1000 cells in two passages. Scale bar: 50 µm.** (I)** Representative FACS analysis showing the ALDH1+ cell populations in SK-3rd and SKOV3 cells.** (J)** The distribution of SKOV3 and SK-3rd cell apoptosis was analyzed by flow cytometry after treatment with cisplatin at a concentration of 20 µM. The proportion of apoptotic and necrotic cells (Annexin V+ and/or PI+) was compared between groups. **(K, L)** Tumor growth curves of nude mice subcutaneously inoculated with serially diluted (10^4^ and 10^5^) SKOV3 or SK-3rd cells (6 mice/group, ***P < 0.001) **(K)**. Representative images of the tumors removed from mice (**L**). The data in B, D, E, H and J are the means ± SD of three independent experiments (*P < 0.05, **P < 0.01 and ***P < 0.001).

To examine the effects of chemotherapy on the stem cell pool in EOC, primary cancer cells were obtained from EOC patients undergoing neoadjuvant chemotherapy (N = 4) or upfront surgery (N = 12), and the proportion of *in vitro* self-renewing cancer cells was compared (**Table S1**). After 14 days of suspension culture, 5×10^3^ Lin- tumor cells from the 4 neoadjuvant chemotherapy patients formed much more spheres than the same number of Lin- cells from the 12 chemotherapy-naive patients (P = 0.027, **Figure 1A-B**). Furthermore, the proportion of CD133+ tumor cells (CD133+Lin- cells) was significantly increased in tumors undergoing neoadjuvant chemotherapy compared with those without preoperative treatment (7.63% *vs.* 0.58%, P = 0.003;** Figure 1C-D**), as well as the proportion of CD44+CD24+Lin- cells (3.85% *vs.* 0.25%, P < 0.0001; **Figure 1C, 1E**). These data suggest that chemotherapy selectively enhances the proportional survival of EOCSCs.

Given that both chemotherapy and suspension culture have been used to expand CSC subpopulations in variety solid tumors and we found supporting clinical evidence that the chemotherapy can result in CSC enrichment in ovarian cancer patients, we attempted to enrich for EOCSCs by consecutively passaging ovarian cancer cells in nude mice treated with chemotherapy in combination with suspension culture *in vitro* (**Figure 1F**).

Cells from the third passage (SK-3rd) were cultured in suspension to generate tumor spheres. The number of spheres reflects the quantity of cells capable of *in vitro* self-renewal 26, 27. A total of 10^3^ SK-3rd cells formed significantly more tumor spheres than 10^3^ SKOV3 cells derived from control mice (P < 0.001, **Figure 1G-H**). A significant difference in sphere formation property was even found between the SK-1st cells and their control SKOV3 cells isolated from the xenografts of the first generation mice, confirming the efficacy of cisplatin treatment for CSC enrichment (**Figure S1A**). Moreover, 58.53% of freshly isolated SK-3rd cells were ALDH+, while 3.37% of SKOV3 cells were ALDH+ (P = 0.002; **Figure 1I, Figure S1B**). In addition, sphere-derived SK-3rd and SK-3rd cells but not control SKOV3 cells highly expressed stem cell-associated genes, such as *BMI-1*, *β-CATENIN*, *SOX-2*, *NANOG*, *KLF-4*, *ABCG2*, *NOTCH1*, *ALDHA1* and *VIMENTIN* 28 (**Figure S1C**). Furthermore, the cell death induced by cisplatin was investigated using flow cytometry. As shown in **Figure 1J**, after treatment with 20 μM cisplatin, the SK-3rd cells had significantly lower proportions of early-apoptotic cells, late-apoptotic and necrotic cells compared to SKOV3 cells (**Figure S1D**).

The tumorigenicity of SK-3rd and SKOV3 cells was examined by a serial xenograft assay in immunocompromised nude mice (**Table S7**). Inoculations of 10^5^ SK-3rd and SKOV3 cells were able to develop xenograft tumors in 6/6 and 3/6 of mice, respectively, and the volumes of the SK-3rd tumors increased faster than those of the SKOV3 tumors (**Figure 1K-L, Figure S1E-F**). Moreover, inoculation with as few as 10^4^ SK-3rd cells resulted in the formation of tumors in all six mice, whereas no tumors were found after 10^4^ SKOV3 cells were administered (**Table S7, Figure 1L and Figure S1E**). Furthermore, IHC of xenografts revealed that tumors from SK-3rd cells exhibited increased expression of Ki-67, ALDH1, EZH2, and VIMENTIN and decreased expression of E-CADHERIN, which suggested that SK-3rd cells not only highly express CSC-associated genes but also may exhibit epithelial-mesenchymal transition (EMT) properties **(Figure S1G).**

### EZH2 knockdown reduces CSCs and inhibits chemoresistance and tumorigenesis in ovarian cancer cells

**Figure 2:**
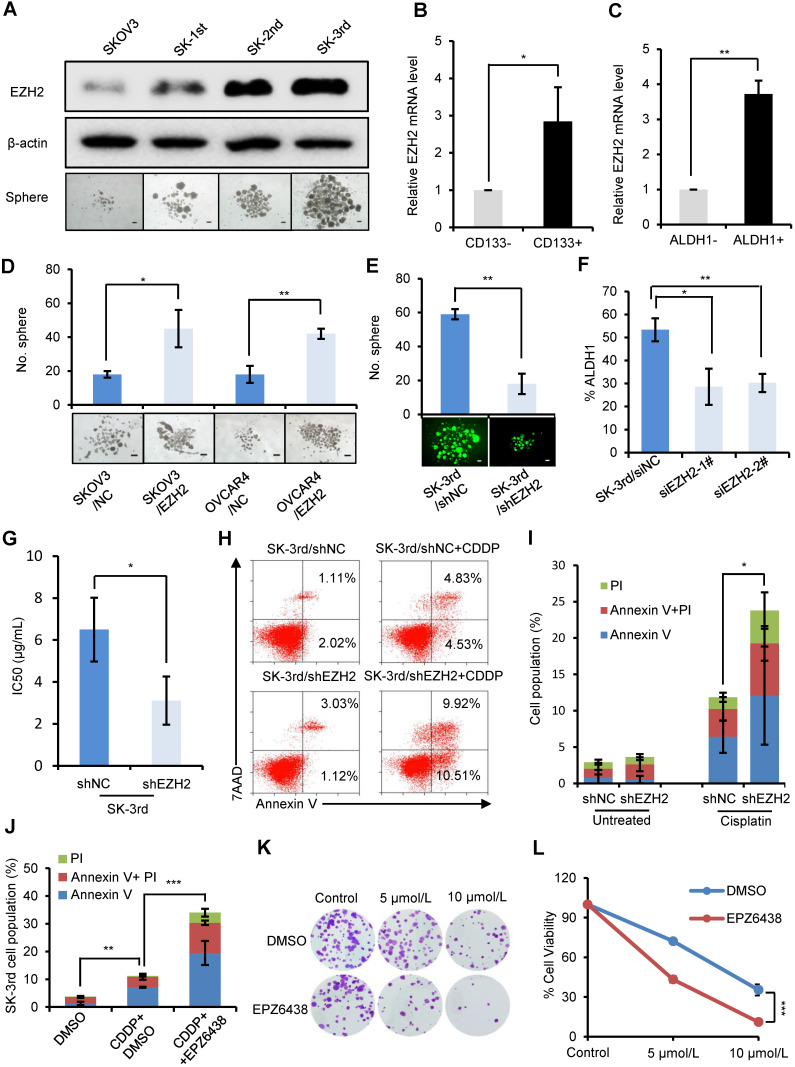
** EZH2 knockdown reduces CSCs and inhibits the chemoresistance and tumorigenesis of ovarian cancer cells. (A)** Western blot analysis of EZH2 expression in SKOV3, SK-1st, SK-2nd and SK-3rd cells (top). Representative images of spheres after 14 days of suspension culture (bottom). The scale bar is 50 µm. **(B, C)** qRT-PCR analysis of *EZH2* mRNA expression in IGROV1/CD133+ **(B)** and SKOV3/ALDH1+ **(C)** cells compared to IGROV1/CD133- or SKOV3/ALDH1- cells. **(D)** Representative images and a statistical histogram of spheres generated by SKOV3 and OVCAR4 cells transfected with an overexpression plasmid DNA for EZH2 or a scrambled control. **(E)** Representative fluorescence images and a statistical histogram of spheres generated by SK-3rd cells transfected with a GFP-tagged lentivirus expressing shRNA targeting EZH2 (shEZH2) or nontargeting scrambled shRNA (shNC). **(F)** The proportion of ALDH1+ cells in SK-3rd cells decreased significantly after genetic knockdown of EZH2. **(G)** The 50% inhibitory concentration (IC50) of cisplatin in SK-3rd/shEZH2 and SK-3rd/shNC cells is shown. **(H, I)** Representative FACS images (**H**) and a statistical histogram (**I**) of the proportion of apoptosis/necrosis in GFP-labeled SK-3rd/shEZH2 cells compared with SK-3rd/shNC cells after 20 µM cisplatin treatment for 48 h. The proportion of apoptotic and necrotic cells (Annexin V+ and/or PI+) was compared between groups. **(J)** Effects of the EZH2 inhibitor (EPZ6438) on apoptosis induction in SK-3rd cells. The cells were treated with DMSO (control), cisplatin (20 µM) plus DMSO, or cisplatin in combination with EPZ6438 (5 µM) for 48 h. The proportion of apoptotic and necrotic cells (Annexin V+ and/or PI+) was compared between groups. **(K, L)** SK-3rd cells were exposed to cisplatin (5 or 10 µM), EPZ6438 (5 µM) or cisplatin in combination with EPZ6438 for 48 h. Representative phase contrast images of colony formation assays (**K**) and the proportion of cell viability (**L**) are shown. The data in B, C, D, E, F, G, I, J and L are the means ± SD of three independent experiments (*P < 0.05, **P < 0.01 and ***P < 0.001).

To examine the effect of EZH2 on CSC populations in ovarian cancer, we first compared the protein level of EZH2 in SKOV3, SK-1st, SK-2nd and SK-3rd cells by Western blot and found a gradual increase in the EZH2 level (**Figure 2A**). Then, we showed that *EZH2* mRNA was significantly increased in IGROV1/CD133+ cells (P = 0.025) and SKOV3/ALDH1+ cells (P = 0.007) compared with IGROV1/CD133- cells and SKOV3/ALDH1- cells, respectively (**Figure 2B-C**). In addition, the effects of EZH2 overexpression and knockdown on spheroid formation in SKOV3, OVCAR4 and SK-3rd cells were evaluated. We found that the number of spheroids generated in SKOV3/EZH2 and OVCAR4/EZH2 cells was significantly greater than that in the control cells (**Figure S2A-B, Figure 2D**). In contrast, SK-3rd/shEZH2 cells generated smaller spheroids than control cells (**Figure S2C, Figure 2E**). Moreover, the proportion of ALDH1+ cells in SK-3rd cells decreased significantly after knocking down EZH2 (**Figure 2F, Figure S2D**). These results indicate that EZH2 contributes to spheroid formation in ovarian cancer cells.

To determine whether the expression of EZH2 is related to platinum resistance, we assessed the viability of SK-3rd/shEZH2 and SK-3rd/shNC cells following treatment with different concentrations of cisplatin and found that SK-3rd/shNC cells had increased resistance to cisplatin (P = 0.037, **Figure 2G, Figure S2E**). Consistently, flow cytometric analysis showed that the proportion of apoptotic/necrotic SK-3rd/shEZH2 cells was significantly increased after treatment with cisplatin at a concentration of 20 μM compared with that of SK-3rd/shNC cells (P = 0.016, **Figure 2H-I**). Additionally, we analyzed the effects of the EZH2 inhibitor EPZ6438 on cisplatin-induced apoptosis and clone formation capacity in SK-3rd cells. The results showed that EZH2 inhibition significantly increased cisplatin-induced cell apoptosis and necrosis (**Figure 2J, Figure S2F**) and reduced the number of colonies formed by SK-3rd cells compared with control groups (**Figure 2K-L**). Finally, the tumorigenicity of SK-3rd cells was found to be undermined by EZH2 depletion. Inoculation with as few as 10^4^ or 10^5^ SK-3rd/shNC cells resulted in the formation of tumors in all mice, whereas no tumors were found after 10^4^ or 10^5^ SK-3rd/shEZH2 cells were administered (**Figure S2G, Table S7**). These results suggest that EZH2 plays a crucial role in maintaining the characteristics of EOCSCs.

### CHK1 is an EZH2 target involved in CSC stemness

**Figure 3:**
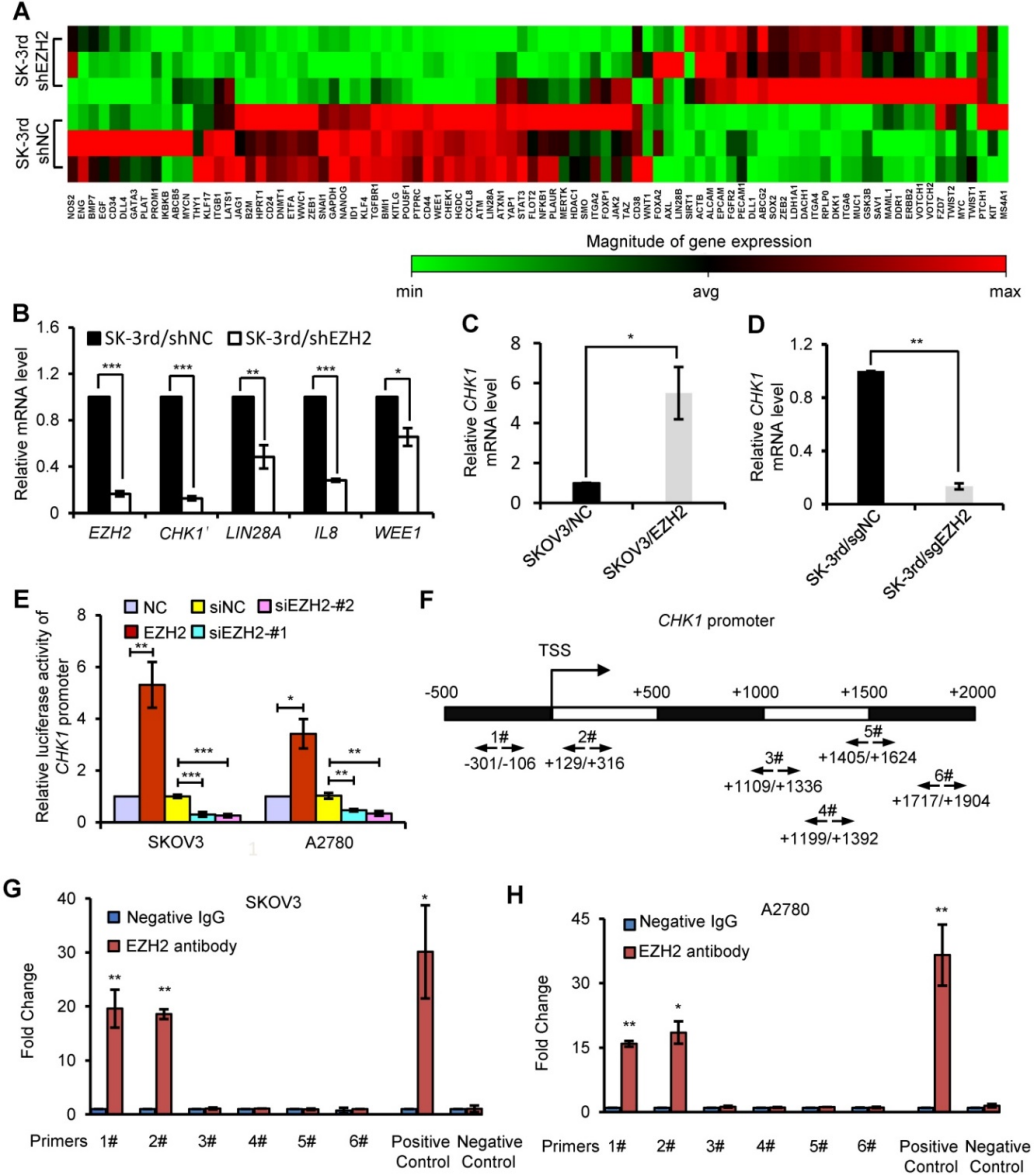
** CHK1 is an EZH2 target involved in CSC stemness. (A)** Hierarchical clustering displays differential expression profiles for SK-3rd/shNC and SK-3rd/shEZH2 cells (N = 3 replicates). Rows represent individual samples, and columns represent genes. Each cell corresponds to the expression level of a particular gene in a given sample. A visual dual color code is used, with red and green indicating relatively high and low expression levels, respectively. A color scale is included to reflect gene expression. **(B)** Relative mRNA levels of *CHK1*, *LIN28A*, *IL8* and *WEE1* in SK-3rd cells transfected with EZH2-targeted shRNA or scrambled shRNA. **(C, D)** qRT-PCR analysis of *CHK1* mRNA expression in SKOV3/EZH2 **(C)** and SK-3rd/sgEZH2 cells **(D)** compared with scrambled control cells. Expression levels were normalized to β-actin. EZH2 was knocked out of SK-3rd cells using the CRISPR-Cas9 system. **(E)** Luciferase activity assays in SKOV3 and A2780 cells showing transactivation of the CHK1 promoter by EZH2 overexpression and repression of the CHK1 promoter by EZH2 silencing. **(F-H)** Schematic illustration of PCR-amplified fragments of the CHK1 promoter that was physically associated with EZH2 **(F)**. Chromatin immunoprecipitation (ChIP) assays were performed using an EZH2 antibody to screen EZH2-bound CHK1 promoter regions (region 1# (nucleotides -301 to -106) and region 2# (nucleotides +129 to +316)) in SKOV3 **(G)** and A2780 **(H)** cells. Human HOXA2 promoter primers were used as positive controls and α-satellite repeat primers were used as negative controls. Blue bars: incubation with negative control IgG; Red bars: incubation with EZH2 antibody. Each negative IgG control was normalized to the unit “1”. The data in B, C, D, E, G and H are the means ± SD of three independent experiments (*P < 0.05, **P < 0.01 and ***P < 0.001).

To identify critical genes and pathways that mediate the effect of EZH2 on EOCSCs, we compared the expression profiles of SK-3rd/shEZH2 and SK-3rd/shNC cells using a CSC-focused PCR array. Unsupervised hierarchical clustering and analysis of variance-based statistical analysis identified that 11 genes were upregulated > 2-fold, while 26 genes were downregulated > 2-fold in SK-3rd/shEZH2 relative to SK-3rd/shNC cells (P < 0.05, **Figure 3A**). Notably, Checkpoint kinase 1 (*CHK1*) was one of the most significantly downregulated genes in SK-3rd/shEZH2 cells compared with SK-3rd/shNC cells (8.79-fold, P < 0.0001; **Figure 3A, Figure S3A**). Moreover, several significantly downregulated genes in SK-3rd/shEZH2 cells were further validated by qRT-PCR (**Figure 3B**). In addition, the interactions between proteins encoded by these downregulated genes and EZH2 were depicted using the STRING database (**Figure S3B**), and the results suggested that CHK1 may be a functional downstream protein of EZH2. Further study based on the mRNA expression data in GSE9891, GSE14764, GSE26712 and TCGA RNAseq V1 identified that *EZH2* was positively associated with the expression of *CHK1* (**Figure S3C**). Moreover, based on the online Kaplan-Meier plotter analysis of different GEO data, the PFS time and the OS time of patients with high CHK1 expression were significantly shorter than those of patients with low CHK1 expression (**Figure S3D-E**).

To further confirm the relationship between EZH2 and CHK1, we manipulated EZH2 expression and assessed CHK1 mRNA and protein levels in EOC cells. CHK1 expression was upregulated in *EZH2*-transduced SKOV3 cells at the mRNA and protein levels but downregulated in *EZH2*-knockout SK-3rd cells compared with control cells (**Figure 3C-D, Figure S3F-I**). Given that the canonical role of EZH2 is its function as an essential component of PRC2 to catalyze the trimethylation of K27 in histone H3 and induce epigenetic silencing of target genes, we analyzed the expression of CHK1 in cells with knockdown of EED or SUZ12, two core components of PRC2, to determine whether CHK1 is a PRC2-dependent target of EZH2. We found no significant difference in CHK1 expression in SK-3rd cells with either EED knockdown or SUZ12 knockdown compared with the control (**Figure S4A-F**). Moreover, our data show that H3K27me3 protein was up-regulated by ectopic expression of wild-type EZH2, but not affected by a catalytically inactive EZH2 mutant lacking the SET domain (ΔSET) in SK-3rd/sgEZH2 cells. However, EZH2 ΔSET mutant increased CHK1 level as efficiently as wild-type EZH2 (**Figure S4G**), suggesting that the histone methyltransferase activity may not be required for CHK1 activation. Consistently, GSK126 or EPZ6438, an inhibitor for EZH2 methyltransferase activity, did not affect CHK1 transcription levels (**Figure S4H**). These findings suggest that CHK1 expression is regulated by EZH2 through a PRC2-independent pathway.

To further validate that CHK1 is transcriptionally regulated by EZH2, a luciferase reporter assay was performed, which showed that the luciferase activity driven by the CHK1 promoter was increased in EZH2-transduced cells but decreased in EZH2-silenced cells in both SKOV3 and A2780 cells (**Figure 3E**). In addition, ChIP assays showed that EZH2 bound directly to region 1 and region 2 within the CHK1 promoter region in both SKOV3 and A2780 cells (**Figure 3F-H**), indicating that EZH2 upregulated CHK1 expression by targeting the CHK1 promoter.

### CHK1 upregulation confers increased cisplatin resistance in OCSCs

**Figure 4:**
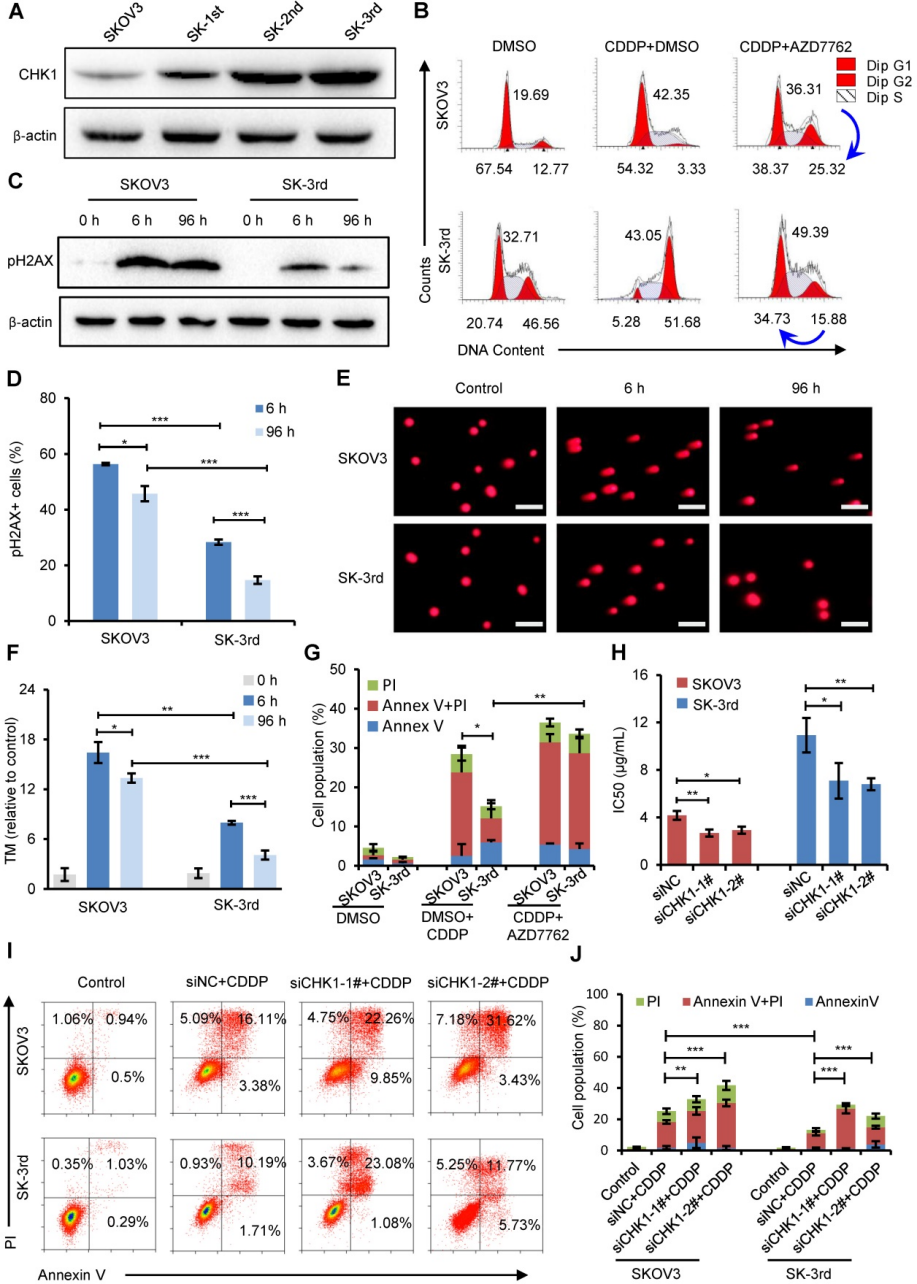
** CHK1 upregulation promotes cisplatin resistance in CSCs. (A)** Western blot analysis of CHK1 levels in SKOV3, SK-1st, SK-2nd and SK-3rd cells. **(B)** Representative FACS images of the cell cycle distributions of SKOV3 and SK-3rd cells treated with DMSO (control), cisplatin (20 µM) plus DMSO, or cisplatin in combination with AZD7762 (5 nM) for 48 h. **(C)** Western blot analysis of pH2AX expression in SKOV3 and SK-3rd cells after 6 h and 96 h of cisplatin treatment. **(D)** Statistical histogram of the proportion of pH2AX-positive cells among SKOV3 and SK-3rd cells after 6 h and 96 h of cisplatin treatment and analysis by immunofluorescence assay. **(E, F)** SKOV3 and SK-3rd cells were exposed to cisplatin for 6 h or 96 h. DNA damage in these cells was measured using a neutral comet assay **(E)**, and tail moment values are shown **(F)**. The scale bar is 100 µm. **(G)** The proportions of apoptotic and necrotic cells among SKOV3 and SK-3rd cells treated with DMSO (control), cisplatin (20 µM) plus DMSO, or cisplatin in combination with AZD7762 (5 nM) for 48 h. The proportion of apoptosis and necrosis cells (Annexin V+ and/or PI+) was compared between groups. **(H)** The IC50 for cisplatin in SK-3rd/siCHK1 *vs.* SK-3rd/siNC cells and that of SKOV3/siCHK1 *vs.* SKOV3/siNC cells. **(I, J)** Representative FACS images **(I)** and a statistical histogram **(J)** of the proportions of apoptotic/necrotic cells among SKOV3 and SK-3rd cells transfected with *CHK1* siRNAs or control siRNA and exposed to 20 µM cisplatin for 48 h. The proportion of apoptotic and necrotic cells (Annexin V+ and/or PI+) was compared between groups. The data in D, F, G, H and J are the means ± SD of three independent experiments (*P < 0.05, **P < 0.01 and ***P < 0.001).

To examine the effect of CHK1 on CSC populations in ovarian cancer, we first compared the CHK1 protein level in SKOV3, SK-1st, SK-2nd and SK-3rd cells and found that the level of CHK1 was gradually increased (**Figure 4A**). Then, we used cell cycle analysis to identify the function of CHK1 in the regulation of ovarian CSCs after treatment with cisplatin. As shown in **Figure 4B**, the proportion of SK-3rd cells in the S phase and the G2/M phase was significantly increased after cisplatin treatment at a concentration of 20 μM compared with those of SKOV3 cells. These data indicate that CSCs were more prone to triggering cell cycle arrest, and this phenomenon was partially reversed by the CHK1 inhibitor AZD7762 (**Figure 4B, Figure S5A**).

Cell cycle arrest has been reported to occur following DNA damage and checkpoint activation 29. One of the earliest modifications to the chromatin structure in the damage response is phosphorylation of histone H2A.X at Ser 139 (pH2AX) 30. We found that short exposure (6 h) of SKOV3 and SK-3rd cells to cisplatin resulted in a considerable increase in pH2AX (**Figure 4C**). However, after 96 h, the pH2AX level in SK-3rd cells was not detectable or only slightly evident but was still high in SKOV3 cells (**Figure 4C**). Similarly, according to the immunofluorescence experiments, we found that the percentage of pH2AX-positive cells in SK-3rd cells was significantly lower than that in SKOV3 cells after 96 h of cisplatin treatment (**Figure 4D, Figure S5B**). We further used a neutral comet assay to detect whether SK-3rd cells are more effective than SKOV3 in repairing cisplatin-induced DNA double-strand damage. As shown in **Figure 4E** and** Figure 4F**, SK-3rd cells had lower tail moment values compared to SKOV3 cells after 6 h of cisplatin treatment; however, after 96 h of cisplatin treatment, the tail moment values in SK-3rd cells were markedly decreased but were still high in SKOV3 cells. Collectively, these results indicated that SK-3rd cells more effectively repaired chemotherapy-induced DNA double-strand damage than SKOV3 cells.

Furthermore, flow cytometry showed that the proportion of apoptotic and necrotic SK-3rd cells treated with 20 μM cisplatin for 48 h was significantly lower than that of SKOV3 cells. However, there was no significant difference between SKOV3 and SK-3rd cells after treatment with AZD7762 (**Figure 4G, Figure S5C**).

To further confirm the correlation between CHK1 and chemoresistance in CSCs, the sensitivities of SKOV3/siNC, SKOV3/siCHK1, SK-3rd/siNC and SK-3rd/siCHK1 cells to cisplatin were assessed by MTT assay and flow cytometry. SKOV3/siCHK1 and SK-3rd/siCHK1 cells exhibited a left-shifted dose-survival curve and became more sensitive to cisplatin than the control cells (**Figure S5D-G**). Compared with the IC50 values of the corresponding control cells, the IC50 values for cisplatin were lower in the SKOV3/siCHK1 and SK-3rd/siCHK1 cells (**Figure 4H**). Similarly, compared with that of the siNC cells, the total proportion of apoptotic and necrotic siCHK1-1^#^ and siCHK1-2^#^ cells was significantly increased in SKOV3 and SK-3rd cells after treatment of cisplatin (**Figure 4I-J**).

We further determined the levels of apoptotic proteins to clarify the effects of CHK1 inhibitor on apoptosis in SK-3rd cells. The results indicated that compared to cisplatin alone, additional AZD7762 treatment significantly increased the cleaved caspase-3 and cleaved caspase-9 levels in SK-3rd cells, but decreased the level of the anti-apoptotic protein BCL-2 (**Figure S5H**). Moreover, ATR, the main kinase responsible for CHK1 phosphorylation and activation under DNA damage, was also significantly downregulated in SK-3rd/sgEZH2 relative to SK-3rd/sgNC cells at both the gene and protein levels (**Figure S6A-B**). AZD6738, an ATR inhibitor, could largely decrease the SK-3rd cells arrested in the S and G2/M phases and increase the SK-3rd cell apoptosis and necrosis after exposure to cisplatin (**Figure S6C-D**). These results suggest that inhibition of CHK1 expression or activity increases the sensitivity of EOCSCs to cisplatin.

In addition to CHK1, ATM was among the genes that were downregulated in EZH2 knockdown cells (**Figure S3A**). Since ATM is one of the main checkpoint kinases, the effects of the ATM inhibitor KU-60019 on the cell cycle and apoptosis in SK-3rd cells treated with cisplatin were tested by flow cytometry. Compared to cisplatin alone, additional KU-60019 led to milder cell cycle arrest in the S and G2/M phases and increased cell deaths but was less effective than the CHK1 inhibitor or ATR inhibitor (**Figure S6E-F**).

### EZH2 promotes CSC properties and chemoresistance by upregulating CHK1

**Figure 5:**
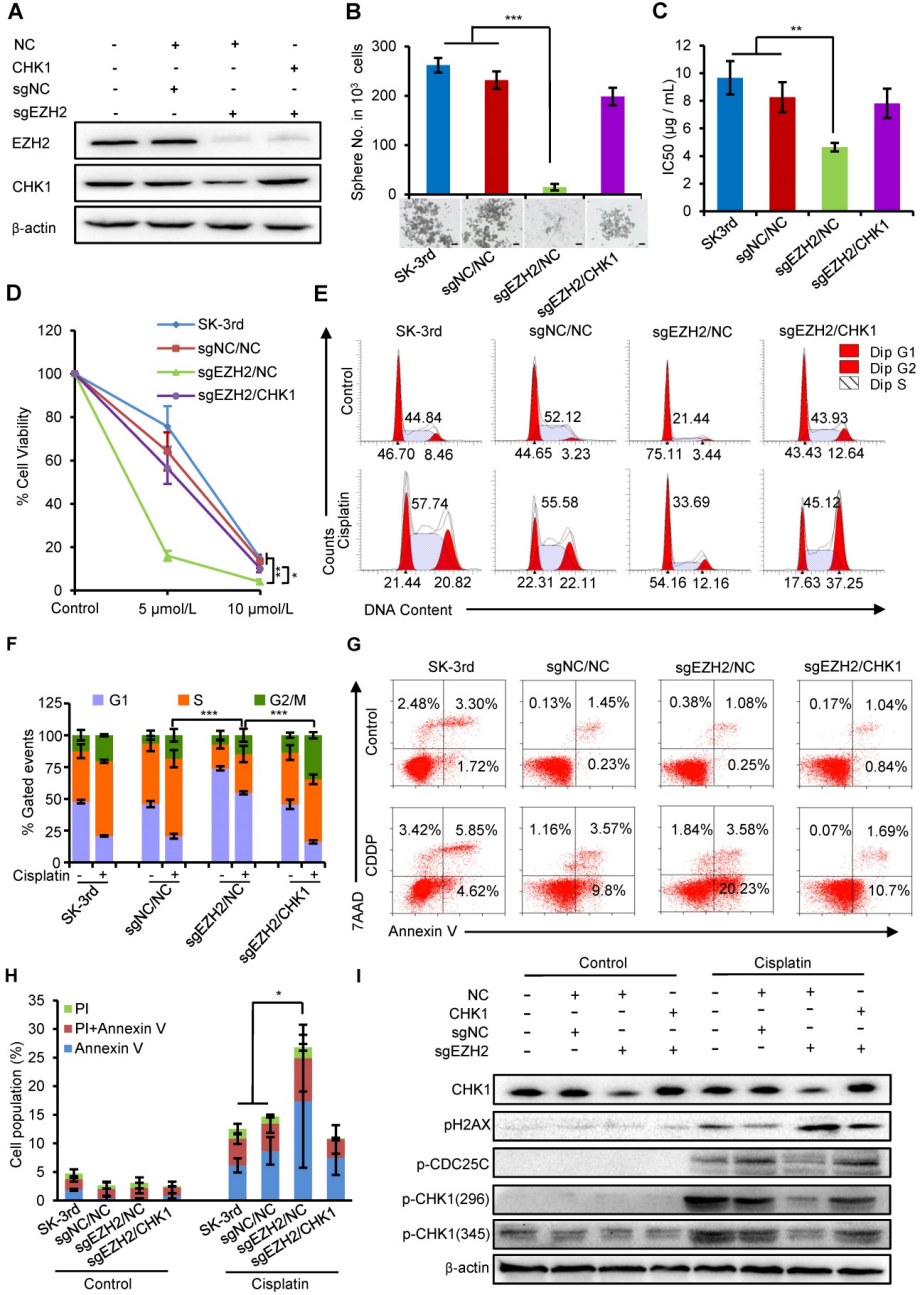
** EZH2 regulates ovarian CSC properties through upregulation of CHK1. (A, B)** Western blot analysis of EZH2 and CHK1 levels in SK-3rd/sgNC and SK-3rd/sgEZH2 cells transfected with an overexpression plasmid for CHK1 or a scrambled control **(A)**. Representative images and the number of spheres formed by 1,000 cells in each group are shown **(B)**. Scale bar: 50 µm. **(C)** The IC50s of cisplatin in SK-3rd, sgNC/NC, sgEZH2/NC and sgEZH2/CHK1 cells are presented as the means ± SD. **(D)** Colony formation assay showing the proportion of cell viability in SK-3rd, sgNC/NC, sgEZH2/NC and sgEZH2/CHK1 cells after cisplatin treatment at a concentration of 5 or 10 µM. **(E, F)** Representative FACS images **(E)** and a statistical histogram **(F)** of the proportions of the cell cycle distributions of SK-3rd, sgNC/NC, sgEZH2/NC and sgEZH2/CHK1 cells treated with PBS (control) or cisplatin (20 µM) for 48 h. The proportion of SK-3rd cells in the S and G2/M phases was compared between groups. **(G, H)** Representative FACS images **(G)** and a statistical histogram **(H)** of the proportions of apoptotic/necrotic cells among SK-3rd, sgNC/NC, sgEZH2/NC and sgEZH2/CHK1 cells treated with PBS (control) or cisplatin (20 µM) for 48 h. The proportion of apoptotic and necrotic cells (Annexin V+ and/or PI+) was compared between groups. **(I)** Western blot analysis for CHK1, pCHK1, pH2AX and p-CDC25C in SK-3rd, sgNC/NC, sgEZH2/NC and sgEZH2/CHK1 cells treated with PBS (control) or cisplatin (20 µM) for 48 h. The data in B, C, D, F and H are the means ± SD of three independent experiments (*P < 0.05, **P < 0.01 and ***P < 0.001).

We next examined whether the effects of increased EZH2 on promoting self-renewal and cisplatin resistance in EOCSCs were partially attributable to CHK1. First, we identified that the protein levels of EZH2 and CHK1 were both downregulated in SK-3rd cells with EZH2 knockout using CRISPR-Cas9 technology (sgEZH2). However, the expression of CHK1 protein was partially restored after upregulating CHK1 with simultaneous downregulation of EZH2 (**Figure 5A**). Our data showed that EZH2 depletion reduced the sphere formation capacity (**Figure 5B**), the IC50 value of cisplatin (**Figure 5C, Figure S7A**) and the number of colonies formed in SK-3rd cells compared with the controls (**Figure 5D, Figure S7B**), while these inhibitory effects were effectively rescued by CHK1 upregulation in SK-3rd/sgEZH2 cells (**Figure 5B-D**).

In addition, PI staining to monitor the cell cycle of SK-3rd/sgEZH2 cells revealed a marked decrease in cell cycle arrest in both the S and G2/M phases compared with that in the control cells after cisplatin treatment (**Figure 5E-F**), which was accompanied by a robust increase in apoptosis (**Figure 5G-H**). Upregulation of CHK1 partially relieved these effects on the cell cycle and apoptosis caused by EZH2 depletion (**Figure 5E-H**).

Finally, we identified that EZH2 depletion largely diminished DNA damage-induced phosphorylation of CHK1 and its downstream protein CDC25C but had no or a slight effect on cells without cisplatin treatment. Similarly, this effect was partially reversed by upregulation of CHK1 (**Figure 5I**). Consistent with increased apoptosis and the loss of DNA damage checkpoints, we also observed increased pH2AX phosphorylation in EZH2-depleted cells, suggesting that EZH2 depletion attenuates activation of the CHK1-CDC25 pathway in response to chemotherapy-induced DNA damage in EOCSCs, which can be rescued by upregulation of CHK1.

### EZH2 expression is positively correlated with the pCHK1 level in human EOC

To further investigate whether EZH2-mediated upregulation of CHK1 exists in human EOCs, we examined 44 paraffin-embedded EOC samples. According to the IHC staining scores, we divided the patient cohort into EZH2 ^low^ vs. EZH2 ^high^ and pCHK1 ^low^ vs. pCHK1^ high^ groups.

**Figure 6:**
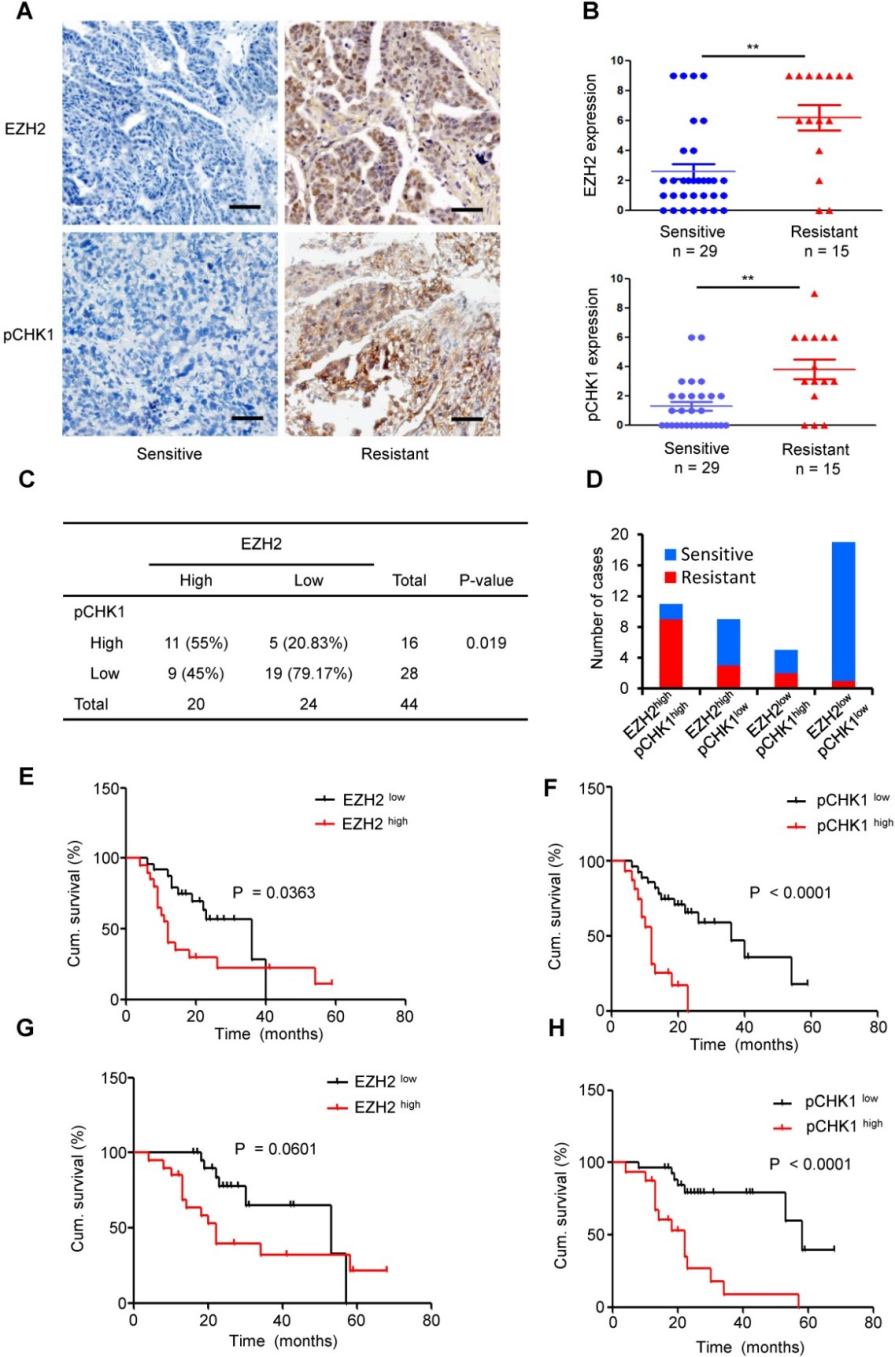
** Overexpression of EZH2 is positively correlated with enhanced expression of pCHK1 in aggressive human ovarian tumors. (A)** EZH2 and pCHK1 expression in EOC tissues taken from 44 patients was assessed by IHC. Representative IHC images of varying EZH2 (top) and pCHK1 (bottom) expression in human EOC are shown. Scale bar: 50 µm. **(B)** The expression of EZH2 or pCHK1 in platinum-resistant ovarian cancer tissues was significantly increased compared with that in platinum-sensitive ovarian cancer tissues. **P < 0.01. **(C)** Pearson's Chi-square test showing that EZH2 expression was associated with pCHK1 expression. **(D)** EZH2^high^/pCHK1^high^ tumors were more likely to be platinum-resistant, whereas EZH2^ low^/pCHK1^ low^ tumors were more likely to be platinum-sensitive. **(E-H)** Kaplan-Meier curves showing the PFS **(E, F)** and OS **(G, H)** of 44 EOC patients stratified by EZH2 or pCHK1 expression status. The log-rank test P values are shown.

High EZH2 and pCHK1 were significantly associated with chemoresistance, relapse and death among patients (**Table S6**). The expression of EZH2 or pCHK1 in platinum-resistant ovarian cancer tissues was significantly increased compared with that in platinum-sensitive tissues (**Figure 6A-B**). In addition, we found that high EZH2 was correlated with an elevated level of pCHK1 (**Figure 6C**, P = 0.019). Of the 44 EOC tumors, 25% (11 patients) fell into the EZH2^high^/pCHK1^high^ subgroup. Notably, 81.82% of the EZH2^high^/pCHK1^high^ tumors were platinum-resistant, whereas 94.74% of the EZH2^low^/pCHK1^low^ tumors were platinum-sensitive (**Figure 6D**).

Logistic analysis revealed that the EZH2^high^ patients had a 2.05-fold increased risk of developing chemoresistance *vs.* EZH2^low^ patients (P = 0.017). Similarly, the risk for chemoresistance was significantly increased by 2.31-fold in the pCHK1^high^ patients compared to the pCHK1^low^ patients (P = 0.005, **Table S8-9**). Moreover, Kaplan-Meier survival analysis indicated that the patients with high EZH2 or pCHK1 expression had significantly shorter PFS (**Figure 6E-F**) and OS (**Figure 6G-H**) than their counterparts.

Finally, both univariate and multivariate Cox regression analyses of PFS were performed for these EOC patients. The variants included age at diagnosis, Federation International of Gynecology and Obstetrics (FIGO) stage, histological subtype, tumor categories, EZH2 expression and pCHK1 expression. The univariate analysis revealed that FIGO stage, EZH2 expression and pCHK1 expression were significantly associated with PFS. In the multivariate analysis, only the pCHK1 level was identified as an independent risk factor for tumor relapse (hazard ratio, 4.26; P = 0.002, **Table S10-11**), indicating that the intratumoral pCHK1 level is a reliable prognostic factor for the PFS of EOC patients.

## Discussion

Our data uncover a previously undescribed role of EZH2 in regulating CHK1-dependent ovarian CSC expansion and show that EZH2 has a critical role in ovarian cancer chemoresistance and progression. EZH2 transcriptionally upregulates CHK1 expression by directly binding to the CHK1 promoter, and inhibition of CHK1abrogates G2/M checkpoints and promotes DNA damaging agent-induced cell death in EOCSC. These results provide strong evidence that targeting EZH2 might be an appealing approach to sensitize EOC to platinum-based chemotherapy.

Increasing evidence has revealed that CSCs are a key population of tumor cells that are highly tumorigenic and chemoresistant in many cancer types 31-33. However, CSC markers are often nonspecific or even unclear, which poses a considerable challenge to targeting these cells. We found that epithelial ovarian tumors from neoadjuvant chemotherapy-treated patients were highly enriched for cells with the properties of CSCs. Therefore, we exploited ovarian CSC chemotherapeutic resistance to generate a highly malignant ovarian cancer cell line (SK-3rd) by sequential *in vivo* passage in cisplatin-treated nude mice. Our data showed that SK-3rd cells display all the defined properties of ovarian CSCs, including an enhanced sphere formation capacity, the ovarian CSC phenotype (ALDH1+) 34, chemotherapy resistance and a strong tumorigenic ability. Among them, SK-3rd cells exhibited higher self-renewal and tumorigenic capacities compared with the SKOV3 cells. In addition, nearly 60% of SK-3rd cells were also ALDH1+, while only a small proportion of the SKOV3 cells were ALDH1+ cells. Collectively, SK-3rd cells are a good model for the study of ovarian CSCs. Similarly, a previous study used the same method to obtain the breast CSCs 35. Therefore, the enrichment and acquisition of large number of CSCs by *in vivo* chemotherapy is a good method for studying CSCs and can be widely used in the studies of other tumors.

EZH2 has emerged as a highly attractive target based on its elevated expression in ovarian cancer and its association with tumor chemoresistance and poor clinical outcomes. In this context, there is a pressing need to identify more specific mechanisms by which EZH2 leads to the development and progression of ovarian cancer. In the present study, we demonstrated that EZH2 is upregulated in ovarian CSCs and sustains the characteristics of CSCs *in vitro* and *in vivo*. Ectopic EZH2 expression increased the sphere formation ability of ovarian cancer cells, whereas EZH2 downregulation in CSCs reduced self-renewal, chemotherapy resistance and tumor-initiating capacity. Our data strengthen those from an earlier study showing that EZH2 acts a critical factor in promoting ovarian cancer chemoresistance and proliferation 36. Similarly, a previous study revealed that EZH2 promotes breast CSC expansion and leads to tumor initiation 37. In addition, it was reported that EZH2 knockdown significantly reduces the frequency of CSCs in pancreatic ductal adenocarcinoma 38. Collectively, these reports are consistent with our study, which showed that EZH2 plays a critical role in regulating CSCs in various tumors.

Despite interest in the association between EZH2 function, ovarian CSCs, and ovarian cancer, the molecular mechanisms underlying the tumorigenic function of EZH2 in ovarian cancer and the relationship with CHK1 signaling have not yet been considered. Emerging data indicate that the link between CHK1 overexpression, tumor recurrence, and therapeutic resistance has been established. A recent report suggested that CHK1 inhibitors, either as single agents or in combination with chemotherapy, represent a viable therapeutic option for the treatment of triple-negative breast cancer 39. In addition, it was reported that some acute myeloid leukemia cells depend on an efficient CHK1-mediated replication stress response for viability, and therapeutic strategies that inhibit CHK1 may extend current cytarabine-based treatments and overcome drug resistance 40. In our study, we also found that overexpression of CHK1 in ovarian CSCs was closely associated with the chemoresistance of ovarian cancer, which is consistent with previous studies. However, despite multiple studies evaluating the association between overexpression of EZH2 or CHK1 and tumor chemoresistance and poor clinical outcomes, the interplay between these two regulators is largely unknown. Thus, whether EZH2 and CHK1 have a direct regulatory relationship warrants attention. Previous studies have found that EZH2 and CHK1 have several common regulatory factors. In breast cancer, Verlinden et al showed that E2F transcription factors can bind to the EZH2 promoter and the CHK1 promoter, suggesting that both EZH2 and CHK1 are physiological targets of the pRB-E2F pathway, which is essential for proliferation and amplification in cancer 41, 42. Additionally, CHK1 is thought to mediate cell cycle arrest in the G2 phase by inhibiting cyclin-dependent kinase 1 (CDK1) 43. Interestingly, another report indicated that CDK1 phosphorylates EZH2 at Thr 487, thereby leading to suppression of H3K27 trimethylation and consequent derepression of target gene expression 44. These studies suggested that loop regulation may occur among the EZH2, CHK1 and CDK1. Moreover, a previous study also demonstrated a link between EZH2 as a regulator of CHK1 phosphorylation in U2OS and HCT116 cancer cells and the DNA damage response 45. Similarly, our study not only identified that EZH2 is a direct regulator of CHK1 expression in ovarian cancer but also demonstrated that CHK1 signal activation is required for EZH2-dependent CSC expansion and maintenance. Furthermore, our data showed that knock down of CHK1 dramatically reduced ovarian CSC survival after cisplatin treatment. The relevance of our findings is further supported by a research study demonstrating that CHK1 inhibitors in combination with chemotherapy dramatically reduced CSC survival *in vitro* by inducing premature cell cycle progression and mitotic catastrophe in non-small cell lung cancer 46. Additionally, another study proved that knockdown of CHK1 successfully inhibited sphere formation of CSCs in the absence of cisplatin in aggressive oral cancer 47. Consistently, we found that upregulation of CHK1 can increase the sphere formation capacity of ovarian CSCs without cisplatin treatment (**Figure 5B**), which may be due to the reduction of DNA damage caused by other external factors.

Many previous studies have shown that the canonical function of EZH2 is mediating trimethylation of histone H3 lysine 27 and inhibiting downstream target genes 10, 48, 49. However, our study demonstrated that down-regulation of EED or SUZ12 had no significant effect on CHK1 expression. Futher study showed that EZH2 ΔSET mutant increased CHK1 level similar to wild-type EZH2, and inhibition of EZH2 methyltransferase activity did not affect CHK1 transcription level, which suggested that the regulation of CHK1 by EZH2 may be independent of PRC-2 function. More recently, several studies showed that EZH2 has a PRC2-independent function in activating downstream genes via methylation of nonhistone targets or direct binding to other proteins 12-15, 50. In our study, we uncovered a mechanism by which EZH2 directly occupies the promoter region of CHK1 and induces its activation in epithelial ovarian cancer, which is consistent with a recent study showing that EZH2 functioned in activating NOTCH1 signaling by directly binding to the NOTCH1 promoter in breast cancer 37.

According to our results, inhibition of CHK1 may increase chemotherapy-induced double-stranded DNA damage and the consequent apoptosis of CSCs. However, little is known about the mechanism. Several recent findings suggested that CHK1 inhibitor-treated human tumor cells hyperactivate ATM, ATR, and caspase-2 after g-radiation and trigger a caspase-2-dependent apoptotic program 51, 52. However, our findings showed that inhibition of CHK1 lead to cisplatin-induced apoptosis of ovarian CSCs, mediated through both caspase-3 and caspase-9 activation. Similar observation had been made in a previous study, which showed that inhibition of CHK1 activated a caspase-3-dependent apoptotic response following DNA replication stress in HCT116 cells 53.

In EOC patient samples, EZH2 and pCHK1 are both significantly overexpressed in samples from platinum-resistant patients compared with those from platinum-sensitive patients. Furthermore, tumors with EZH2 or pCHK1 overexpression were strongly associated with chemoresistance, relapse, and even mortality compared to tumors with low expression of EZH2 or pCHK1. These results supported the suggestion that regulation of the EZH2/CHK1 axis in tumor chemoresistance also occurs *in vivo* and in EOC tissues. More importantly, the expression of EZH2 and pCHK1 is critical for predicting the prognosis of ovarian cancer.

However, this study also has several limitations. First, we mainly focused on the SKOV3 cell line and its derivatives. According to the previous research, although the exact histological origin of SKOV3 is not specified, it is commonly used as a model for high-grade serous ovarian cancer 21. Additionally, SKOV3 cells should serve as a suitable model for studying the sphere-forming ability and tumorigenicity of CSCs. Finally, to compensate for the defects of one cell line, we also added the A2780, OVCAR4, and IGROV1 cell lines and human specimens in some important experiments. Second, we were unable to demonstrate that EZH2 expression is a reliable prognostic factor for the PFS of EOC patients. This shortcoming may be a reflection of inadequate sample size, as the sample was not sufficiently large for small differences to be detected.

In conclusion, our findings establish a previously unrecognized but especially strong connection between EZH2/CHK1 signaling, CSCs and platinum resistance in epithelial ovarian cancer. By providing previously unidentified evidence that EZH2 promotes the platinum resistance of ovarian CSCs by directly activating CHK1 signaling, our work paves the way for targeting EZH2 to reverse recurrence and platinum resistance in ovarian cancer.

## Supplementary Material

Supplementary figures and tables.

## Abbreviations

CDK1: cyclin-dependent kinase 1
ChIP: chromatin immunoprecipitation assay
CHK1: checkpoint kinase 1
CSCs: cancer stem cells
DMSO: dimethyl sulfoxide
EGF: epidermal growth factor
EMT: epithelial-mesenchymal transition
EOC: epithelial ovarian cancer
EOCSCs: epithelial ovarian cancer stem cells
EZH2: enhancer of zeste homolog 2
FGF: fibroblast growth factor
FIGO: federation international of gynecology and obstetrics
GEO: gene expression omnibus website
H3K27me3: trimethylation of lysine 27 on histone 3
IC50: 50% inhibitory concentration
ITS: insulin-transferrinselenium
LIF: leukemia inhibitory factor
OCSCs: ovarian cancer stem cells
OS: overall survival
PFS: progression-free survival
pH2AX: phosphorylation of histone H2A.X at Ser 139
PPI: protein-protein interaction
PRC2: polycomb repressive complex 2
qRT-PCR: real-time quantitative PCR
RT-PCR: reverse transcription-PCR
TCGA: the cancer genome atlas
