# Supplementary material for: EZH2 activates CHK1 signaling to promote ovarian cancer chemoresistance by maintaining the properties of cancer stem cells
Source: Theranostics. 2021 Jan 1;11(4):1795–813. doi: 10.7150/thno.48101 (PMC7778604; doi:10.7150/thno.48101)
Supplement: Supplementary file 1 — Supplementary figures and tables. [file thnov11p1795s1.pdf]

Supplementary figures, legends and tables

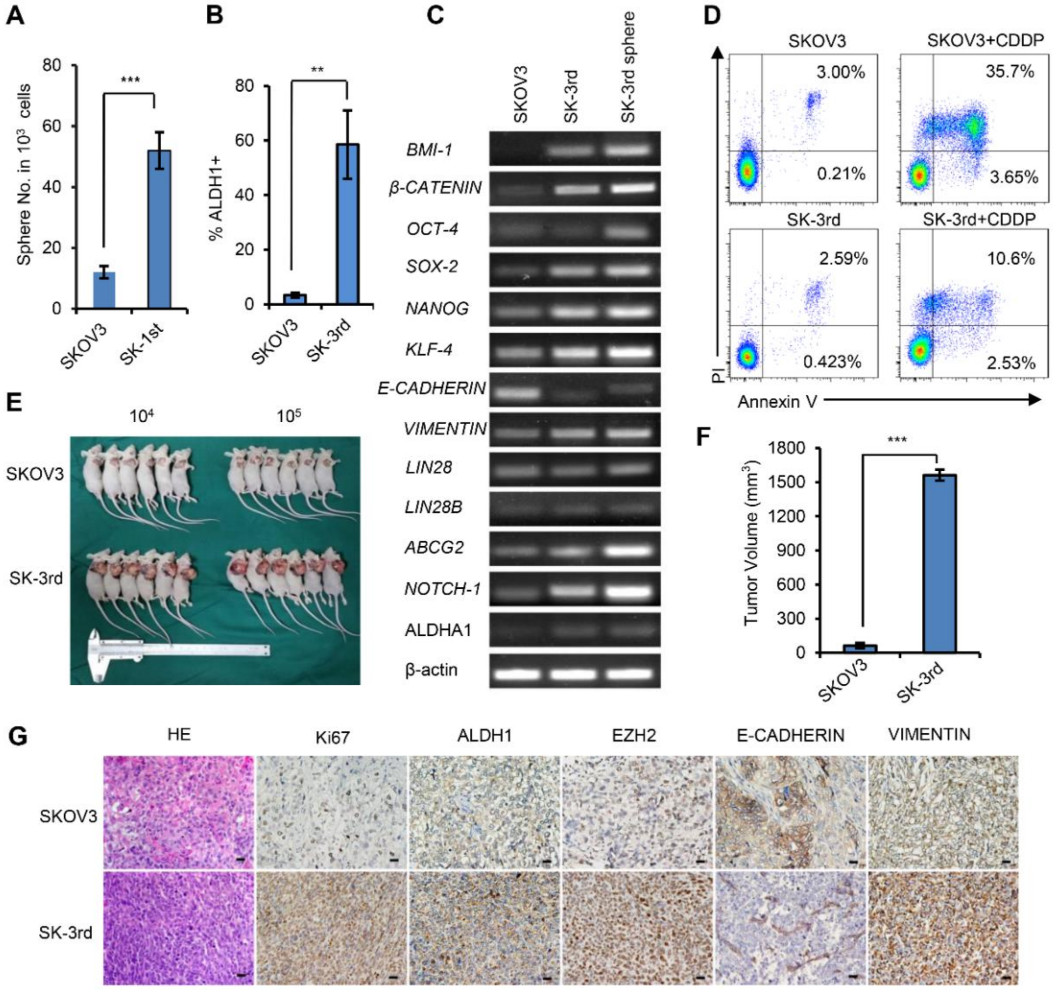

Figure S1. The SK-3rd cells are rich in CSCs compared with SKOV3 cells.

(A) Statistical histogram of the numbers of spheres formed by SK-1st cells compared to their control SKOV3 cells isolated from the xenografts of the first generation mice.

(B) Flow cytometric analysis of the proportion of ALDH1+ cells in SKOV3 and SK-3rd cells (N = 3, $^{**}P < 0.01$). Data represent three independent replicates.

(C) RT-PCR assays revealed that the sphere-derived SK-3rd and SK-3rd cells highly expressed stem cell-associated genes (*BMI-1, β-CATENIN, SOX-2, NANOG, KLF-4*,

ABCG2, *NOTCH1*, *ALDHA1 and VIMENTIN*) compared with the SKOV3 cells.

(D) Analysis of cell apoptosis by flow cytometry assay in SKOV3 and SK-3rd cells treated with 20 μM cisplatin. Numbers in the quadrants represent cells (%) showing the fraction of early apoptotic cells (Annexin V+/PI−) and late apoptotic cells (Annexin V+/PI+).

(E) Representative images of mice bearing SKOV3 and SK-3rd xenograft.

(F) Statistical histogram of the tumor volume of SK-3rd xenografts (10^5) compared to SKOV3 xenografts (10^5) by Student's t test at Day 28.

(G) H&E staining and immunohistochemical staining of the xenografts for Ki-67, ALDH1, EZH2, E-CADHERIN and VIMENTIN.

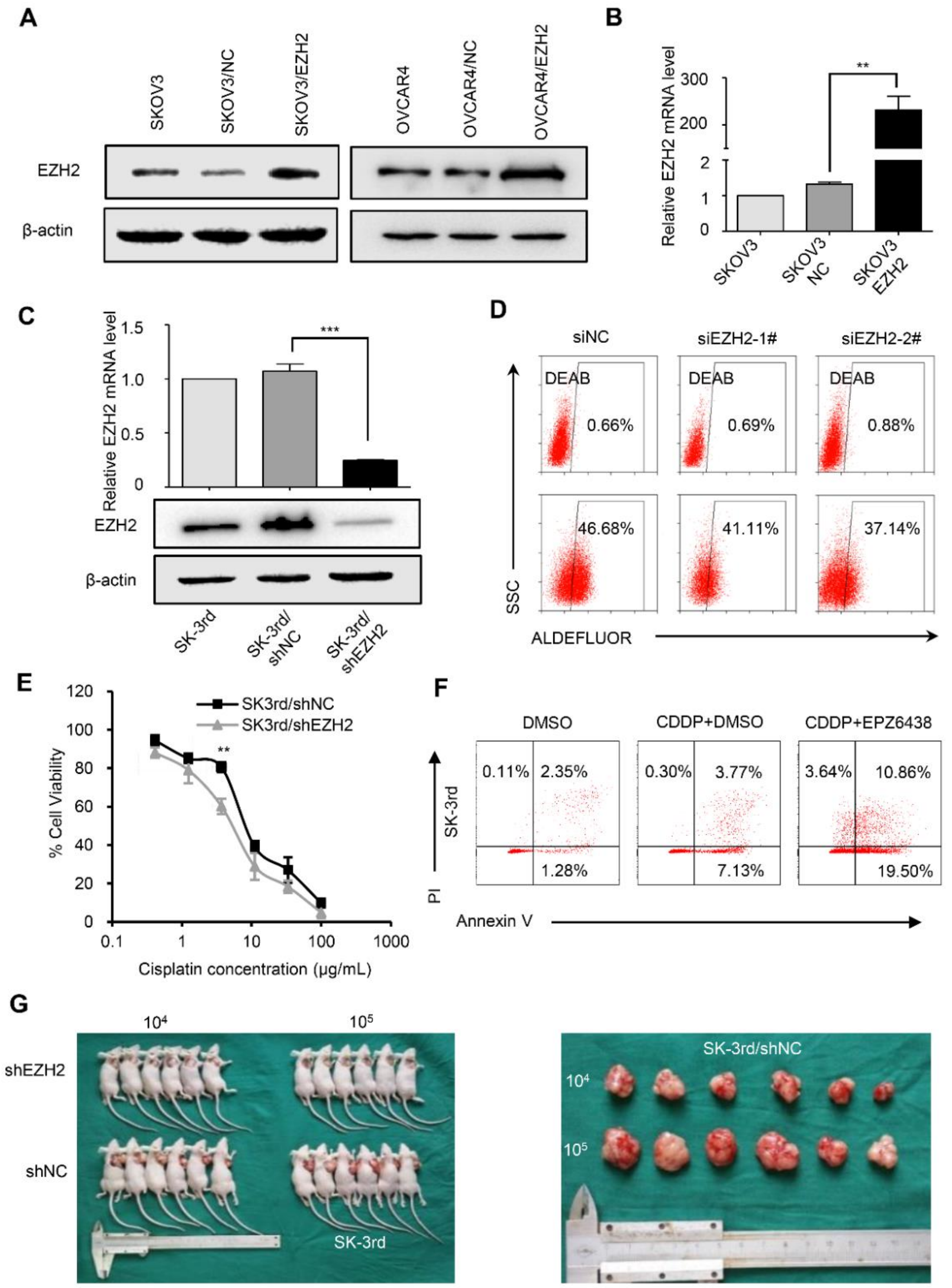

Figure S2. EZH2 knockdown inhibits chemoresistance and tumorigenesis of EOCSCs.

(A, B) Western blotting (A) and qRT-PCR (B) analysis showed that EZH2 levels were dramatically increased in SKOV3/EZH2 and OVCAR4/EZH2 cells compared with the scrambled control cells (**P < 0.01). Data represent of three independent experiments.

(C) Western blotting and qRT-PCR analysis of EZH2 levels in SK-3rd/shEZH2 cells compared with SK-3rd/shNC cells (***P < 0.001). Data represent of three independent experiments.

(D) Flow cytometric analysis showed that siRNA mediated EZH2 knockdown decreased the proportion of SK-3rd/ALDH1+ cells.

(E) The relative viability of SK-3rd/shEZH2 and SK-3rd/shNC cells was measured by MTT assay in triplicate after treating cells with the indicated concentrations of cisplatin (**P < 0.01). The data represent three independent experiments.

(F) Representative FACS images of the proportion of apoptotic/necrotic cells in SK-3rd cells treated with DMSO (control), cisplatin (20 μM) plus DMSO, or cisplatin in combination with EPZ6438 (5 μM) for 48 h.

(G) Nude mice were subcutaneously inoculated with serially diluted (10^4 and 10^5) SK-3rd/shNC or SK-3rd/shEZH2 cells (6 mice/group). Representative images of mice bearing xenograft (Left) and the tumors removed from mice (Right).

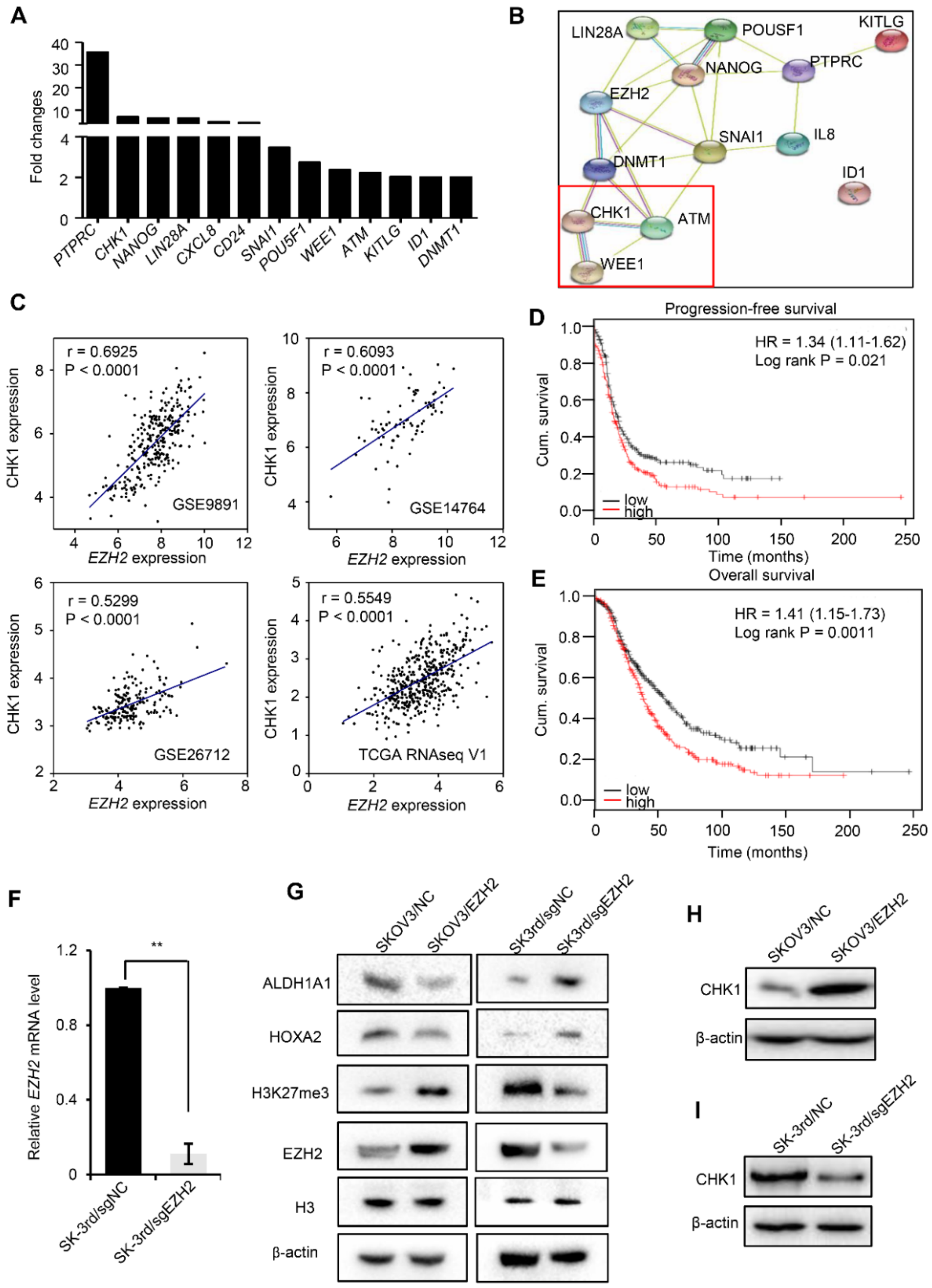

Figure S3. CHK1 is an EZH2 target that is involved in CSC stemness.

(A) PCR microarray analysis showed that the expression of 13 genes was higher in SK-3rd/shNC cells than in SK-3rd/shEZH2 cells.

(B) The interaction between EZH2 and its candidate target proteins are depicted using

STRING 11 database (https://string-db.org/).

(C) Scatter plots showing the positive association between *EZH2* and *CHK1* mRNA levels.

(D, E) Kaplan-Meier curves of progression-free survival (PFS) **(D)** and overall survivals (OS) **(E)** reveal that upregulated CHK1 is associated with poor prognosis in ovarian cancer patients. P-value was obtained by log-rank test.

(F) qRT-PCR analysis of *EZH2* expression in SK-3rd/sgEZH2 cells compared with the scrambled control cells (**P < 0.01). The data represent three independent experiments.

(G) Western blotting confirmed the expression of EZH2 and the identified epigenetic targets of EZH2, including ALDH1A1, HOXA2 and H3K27me3, in SKOV3/EZH2 and SK-3rd/sgEZH2 cells compared with the scrambled control cells.

(H, I) Western blotting analysis of CHK1 expression in SKOV3/EZH2 **(H)** and SK-3rd/sgEZH2 **(I)** cells compared with the scrambled control cells.

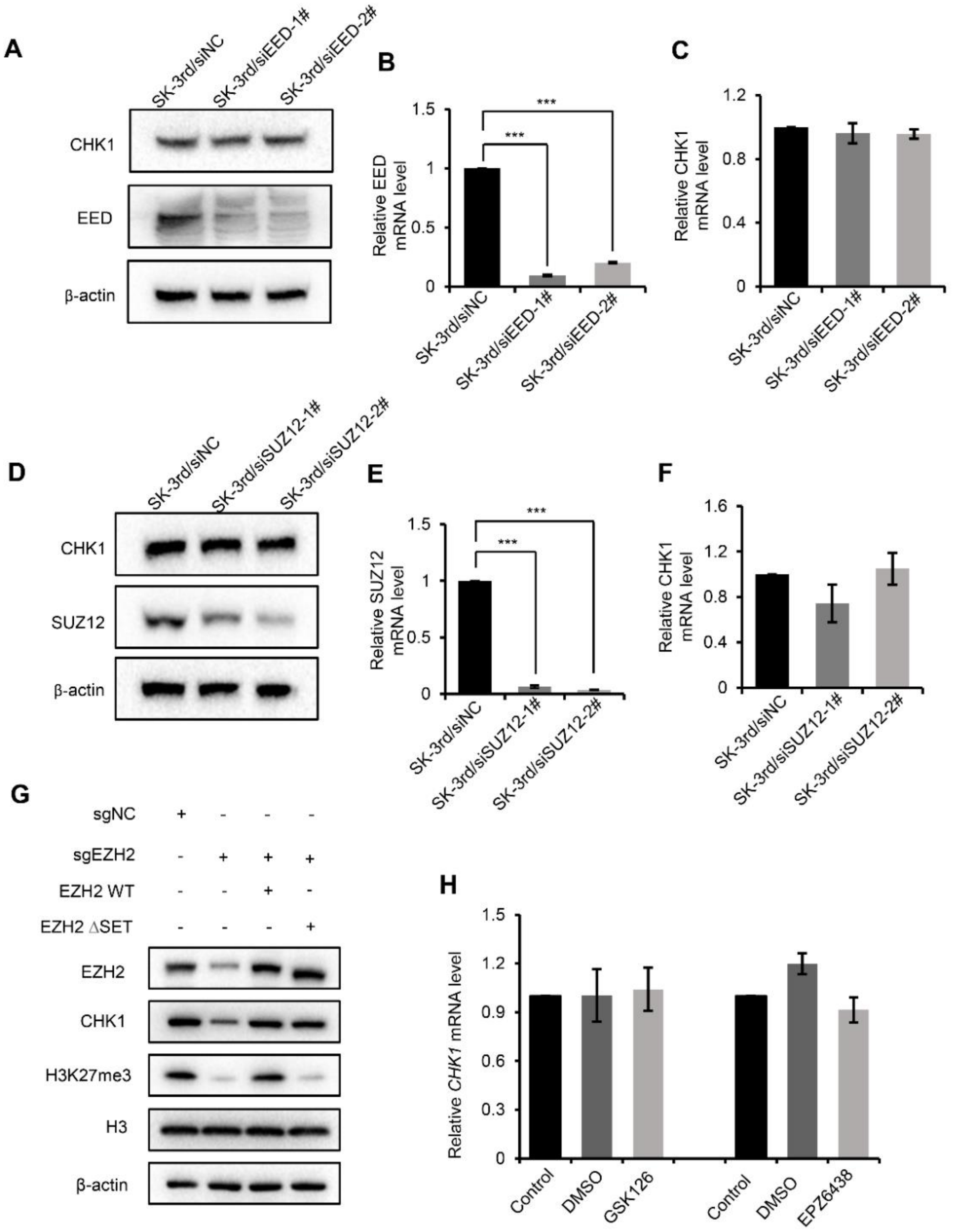

Figure S4. CHK1 is regulated by EZH2 through a PRC2-independent pathway.

(A-C) Western blotting **(A)** and qRT-PCR analysis of EED (**B**) and CHK1 (**C**) levels in SK-3rd cells transfected with EED siRNAs or control siRNA (***P < 0.001). The data represent three independent experiments.

(D-F) Western blotting **(D)** and qRT-PCR analysis of SUZ12 (**E**) and CHK1 (**F**) levels in SK-3rd cells transfected with SUZ12 siRNAs or control siRNA (***P <

0.001). The data represent three independent experiments.

(G) Western blotting analysis of EZH2, CHK1 and H3K27me3 levels in SK-3rd/sgEZH2 cells infected with a retrovirus encoding wild-type EZH2 (WT) or a SET domain-deleted EZH2 mutant (EZH2 ΔSET).

(H) qRT-PCR analysis of CHK1 levels in SK-3rd cells treated with DMSO, GSK126 (2.5 μM) or EPZ6438 (5 μM) for 48 h. The data represent three independent experiments.

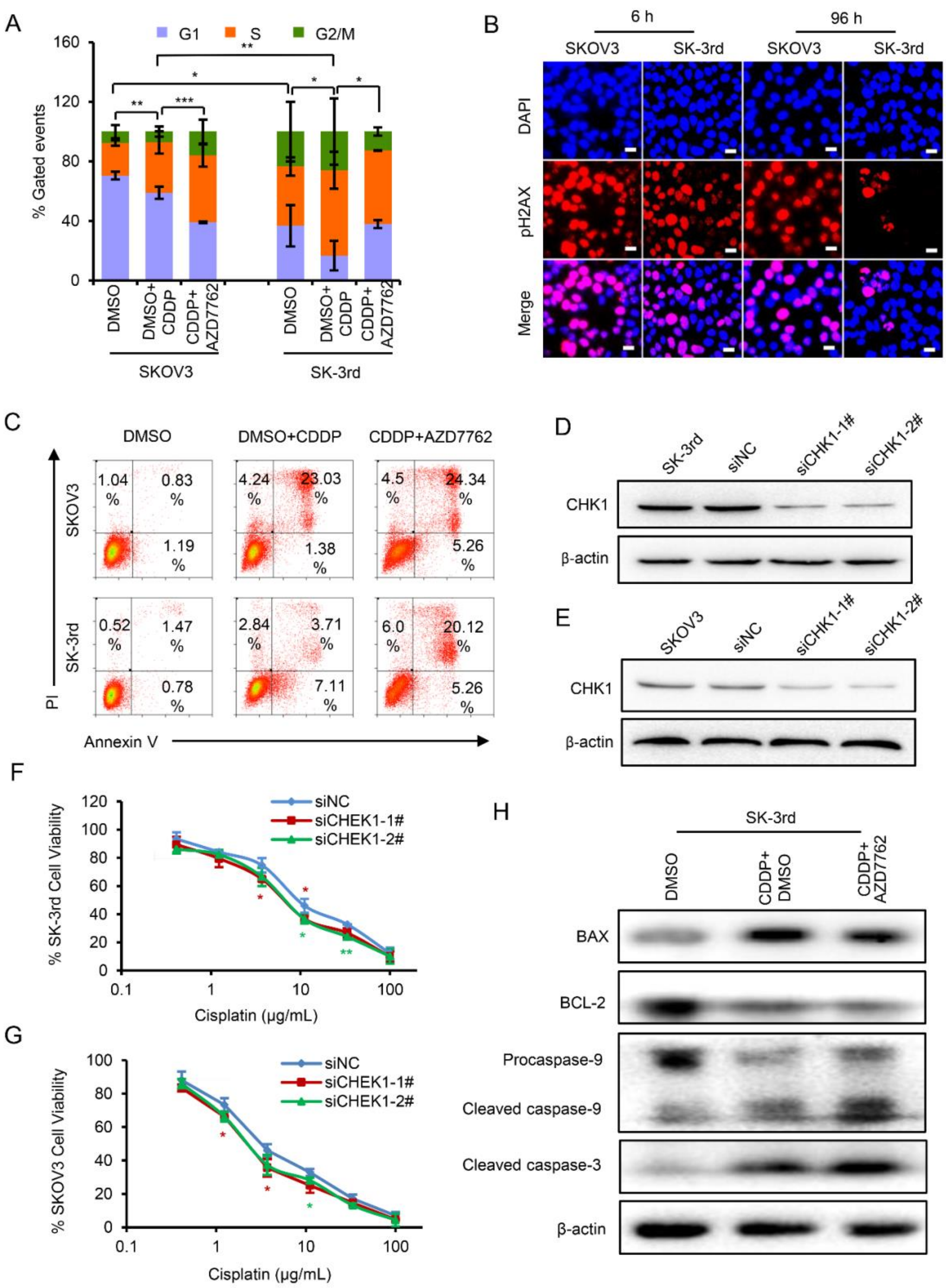

Figure S5. CHK1 downregulation increased cisplatin sensitivity in EOCSCs.

(A) SKOV3 and SK-3rd cells were treated with DMSO (control), cisplatin plus DMSO (20 μM), or cisplatin in combination with AZD7762 (5 nM) for 48 h. The proportion of SK-3rd cells in the S and G2/M phases was compared between groups (*P < 0.05, **P < 0.01 and ***P < 0.001). The data represent three independent

experiments.

(B) The levels of pH2AX were detected by immunofluorescence assay in SKOV3 and SK-3rd cells after 6 h or 96 h cisplatin treatment.

(C) Representative FACS plot showing the results of apoptosis in SKOV3 and SK-3rd cells treated with DMSO (control), DMSO plus cisplatin (20 μM), or cisplatin in combination with AZD7762 (5 nM) for 48 h respectively. Images represent representative of three independent experiments.

(D, E) Western blotting analysis of CHK1 levels in SK-3rd **(D)** or SKOV3 **(E)** cells transfected with CHK1 siRNAs or control siRNA.

(F, G) Cell viability of SK-3rd cells (**F**) and SKOV3 cells (**G**) transfected with CHK1 siRNA or control siRNA (*$P < 0.05$, **$P < 0.01$). The data represent three independent experiments.

(H) Western blotting analysis of the BAX, BCL-2, Cleaved caspase-3 and caspase-9 levels in SK-3rd cells treated with DMSO (control), DMSO plus cisplatin (20 μM), or cisplatin in combination with AZD7762 (5 nM) for 48 h respectively.

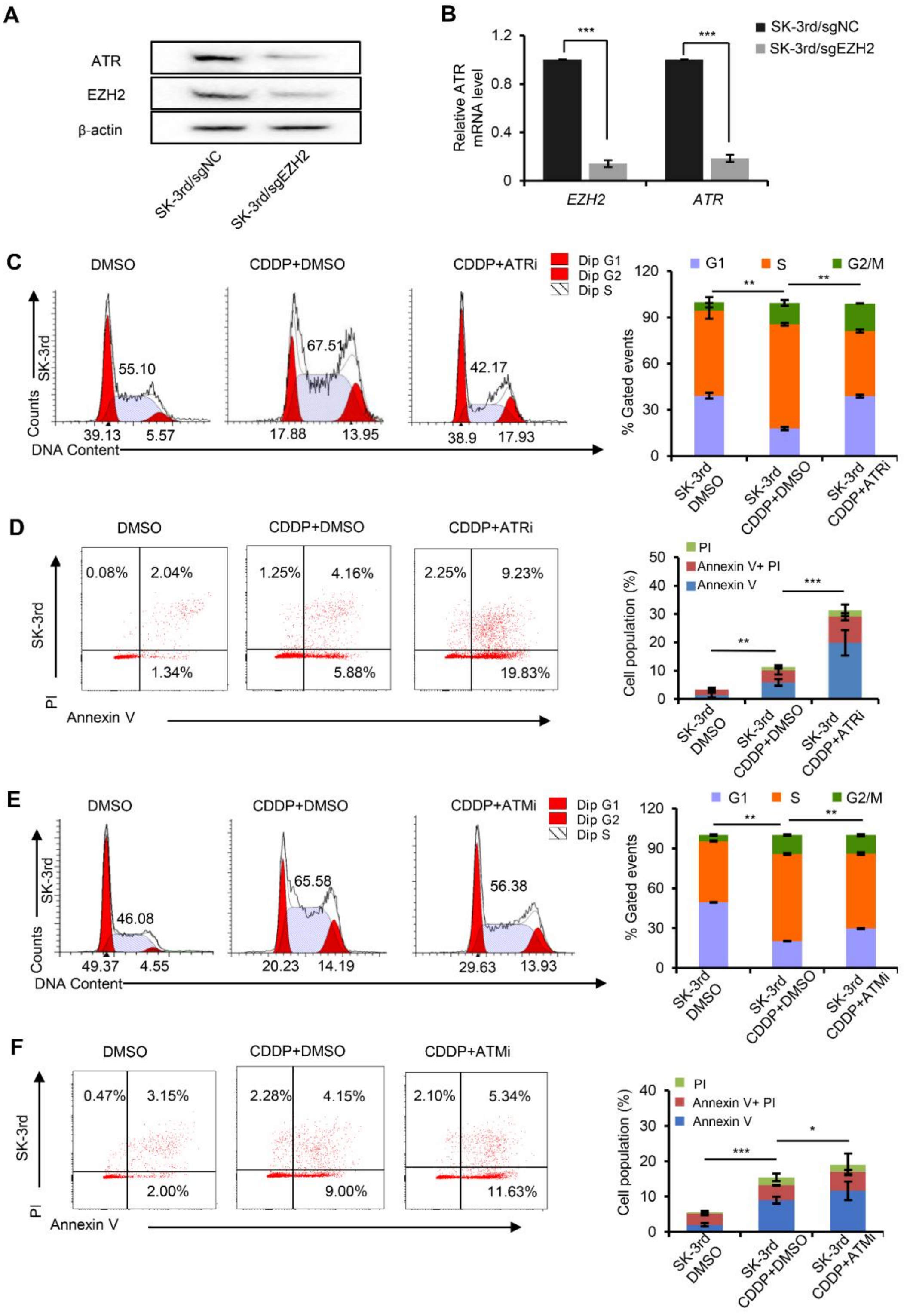

Figure S6. ATR and ATM inhibitors prevent cisplatin-induced cell cycle arrest and promote apoptosis in human EOCSCs.

(A, B) Western blot **(A)** and qRT-PCR **(B)** analysis of ATR and EZH2 levels in SK-3rd/sgEZH2 cells compared with the scrambled control cells (***P < 0.001).

The data represent three independent experiments.

(C, D) Cell cycle distribution **(C)** and apoptosis rate **(D)** of SK-3rd cells treated with DMSO (control), cisplatin plus DMSO (20 μM) or cisplatin in combination with ATRi (5 μM) for 48 h. The proportion of SK-3rd cells in the S and G2/M phases and proportion of apoptotic and necrotic cells (Annexin V+ and/or PI+) were compared between groups (**P < 0.01 and ***P < 0.001). All data represent three independent experiments.

(E, F) Cell cycle distribution **(E)** and apoptosis rate **(F)** of SK-3rd cells treated with DMSO (control), cisplatin plus DMSO (20 μM) or cisplatin in combination with ATMi (2 μM) for 48 h. The proportion of SK-3rd cells in the S and G2/M phases and proportion of apoptotic and necrotic cells (Annexin V+ and/or PI+) were compared between groups (*P < 0.05, **P < 0.01 and ***P < 0.001). All data represent three independent experiments.

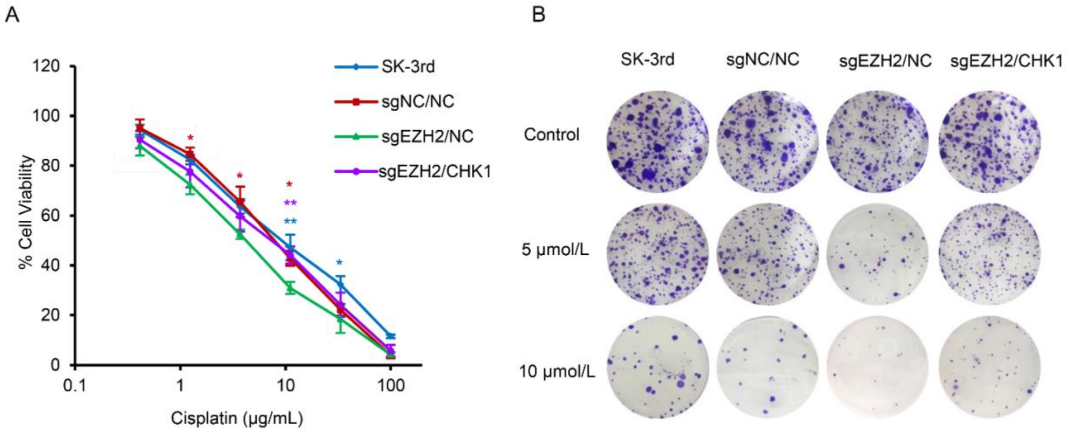

Figure S7. The decreased cisplatin resistance induced by EZH2 depletion can be rescued by the upregulation of CHK1 in EOCSCs.

(A) Cell viability of SK-3rd/sgNC and SK-3rd/sgEZH2 cells transfected with plasmid

expressing CHK1 or the corresponding control plasmid after cisplatin treatment at different concentrations (*$P < 0.05$, **$P < 0.01$). The data represent three independent experiments.

(B) Representative image of colony formation assays from SK-3rd/sgNC and SK-3rd/sgEZH2 cells transfected with plasmid expressing CHK1 or negative control after cisplatin treatment at a concentration of 5 or 10 μM.

Table S1 Patient clinical records and frequency of CSCs.

Patient #	FIGO	Histological grading	Histology	Neoadjuvant chemotherapy	Spheres /5000 cells	Lin-/CD133+ (%)	Lin-/CD24+ /CD44+ (%)	Chemotherapy resistance	Relapse	Death
01	IV	Low	Serous	0	4	0.35	1.29	No	No	No
02	III	Low	Clear cell	0	8	0.63	0.73	No	No	No
03	IV	Low	Serous	TP×3	16	3.0	4.85	No	Yes	Yes
04	II	Low	Serous	TP×3	38	2.31	4.22	No	No	No
05	I	Low	Mucinous	0	11	0.19	0.11	No	No	No
06	IV	Low	Serous	0	8	0.64	0.17	Yes	Yes	No
07	III	Low	Serous	TP×3	34	18.2	5.36	Yes	Yes	No
08	I	High	Mucinous	0	7	0.63	0.26	No	No	No
09	III	Low	Serous	0	10	0.72	0.03	No	No	No
10	I	High	Serous	0	6	1.59	0.23	No	No	No
11	III	Low	Serous	0	4	0.22	0.1	No	No	No
12	III	Low	Serous	0	4	0.79	0.02	No	No	No
13	I	Low	Mucinous	0	3	0.04	0.004	No	No	No
14	I	High	Endometrioid	TP×2	20	7.01	0.96	No	No	No
15	III	High	Serous	0	0	1.1	0.1	No	No	No
16	III	Low	Serous	0	8	0.06	0.03	No	No	No

FIGO: Federation International of Gynecology and Obstetrics. TP: paclitaxel and carboplatin.

Table S2 The siRNA targeting sequences.

Gene name	Forward primer	Reverse primer
siCHK1-1#	GGUUUAUCUGCAUGGUAUUTT	AAUACCAUGCAGAUAAACCTT
siCHK1-2#	GGCAACAGUAUUUCGGUAUTT	AUACCGAAAUACUGUUGCCTT
siEED-1#	GGCCAUGGAAAUGCUAUCATT	UGAUAGCUUUCCAUGGCCTT
siEED-2#	GGUGCUGCUAUUCGACAAATT	UUUGUCGAAUAGCAGCAGCACCTT
siSUZ12-1#	CUGCCGCAAACUUUAUAGUTT	ACUAUAAAGUUUGCGGCAGTT
siSUZ12-2#	GGGACAGAAAUGGAUUUATT	UAAAUCCAUUUGCUGUCCCTT
siNC	AATTCTCCGAACGTGTCACGT	

Table S3 List of antibodies.

Antibodies	Clone	Catalog	Manufacturer	Application(s)
CD133/AC133-PE	AC133	130-080-801	Miltenyi Biotec (GmbH, Germany)	FACS
CD140a-APC	APA5	17-1401-81	eBioscience (San Diego, CA)	FACS
CD235a-APC	HIR2 (GA-R2)	17-9987-42	eBioscience (San Diego, CA)	FACS
CD24-FITC	ebiosn3	11-0247-42	eBioscience (San Diego, CA)	FACS
CD31(PECAM-1)-APC	WM59	17-0319-42	eBioscience (San Diego, CA)	FACS
CD44-PE-CY7	G4426	560533	Becton, Dickinson and company (USA)	FACS
CD45-APC	2D1	17-9459-42	eBioscience (San Diego, CA)	FACS
Mouse IgG Isotype Control-APC	P3.6.2.8.1	17-4714-82	eBioscience (San Diego, CA)	FACS
Mouse IgG Isotype Control-FITC	P3.6.2.8.1	11-4714-81	eBioscience (San Diego, CA)	FACS
Mouse IgG Isotype Control-PE	P3.6.2.8.1	12-4714-41	eBioscience (San Diego, CA)	FACS
EZH2	D2C9	# 5246	Cell Signaling Technology, Beverly, MA	IHC(1:200); WB(1:1000)

Ki67	EPR3610	ab92742	Abcam (Cambridge, UK)	IHC(1:600)
E-CADHERIN	EP913(2)Y	ab76319	Abcam (Cambridge, UK)	IHC(1:200)
ALDH1	Polyclonal-Rabbit	# 15910-1-AP	Proteintech Group, Chicago, USA	IHC(1:1000)
VIMENTIN	EPR3776	ab92547	Abcam (Cambridge, UK)	IHC(1:600)
Goat anti-rabbit Cy3-labeled secondary antibody	-	072-01-15-06-1	KPL, Gaithersburg, MD, USA	IF(1:1000)
CHK1	2G1D5	# 2360	Cell Signaling Technology,Beverly, MA	WB(1:1000)
ALDH1A1	monoclonal-Rabbit	A0157	ABclonal (Boston, USA)	WB(1:1000)
HOXA2	Polyclonal-Rabbit	A9658	ABclonal (Boston, USA)	WB(1:1000)
TriMethyl-Histone H3-K27	Methylated-Rabbit	A2363	ABclonal (Boston, USA)	WB(1:1000)
H3	Polyclonal-Rabbit	A2348	ABclonal (Boston, USA)	WB(1:1000)
EED	Polyclonal-Rabbit	A5371	ABclonal (Boston, USA)	WB(1:1000)
SUZ12	monoclonal-Rabbit	A4348	ABclonal (Boston, USA)	WB(1:1000)
pH2AX(Ser139)	20E3	#9718	Cell Signaling Technology,Beverly, MA	IF(1:400); WB(1:1000)
BAX	E63	ab32503	Abcam (Cambridge, UK)	WB(1:1000)
BCL-2	E17	ab32124	Abcam (Cambridge, UK)	WB(1:1000)
Caspase9	-	#9502S	Cell Signaling Technology,Beverly, MA	WB(1:1000)
Cleaved caspase-3	5A1E	#9664S	Cell Signaling Technology,Beverly, MA	WB(1:1000)
ATR	E1S3S	# 13934	Cell Signaling Technology,Beverly, MA	WB(1:500)
pCHK1 (S345)	Polyclonal	ab47318	Abcam (Cambridge, UK)	IHC(1:200)
pCHK1 (S345)	133D3	# 2348	Cell Signaling Technology,Beverly, MA	WB(1:200)
pCHK1(296)	EPR915	# ab79758	Abcam (Cambridge, UK)	WB(1:1000)

Phospho-cdc25C (Ser216)	-	#9528	Cell Signaling Technology,Beverly, MA	WB(1:500)
Peroxidase-labeled goat anti-mouse secondary antibody	-	474–1806	KPL, Gaithersburg, MD, USA	WB(1:5000)
Peroxidase-labeled goat anti-rabbit secondary antibody	-	474–1516	KPL, Gaithersburg, MD, USA	WB(1:5000)
Biotinylated goat anti-rabbit secondary antibody	-	SP-9001	ZSGB-BIO, Beijing, China	IHC
Rabbit IgG Isotype Control	Polyclonal	ab37415	Abcam (Cambridge, UK)	IHC(1:200)

Table S4 Primer sequences of target genes.

Gene name	Upstream	Downstream	T_m
EZH2	TTGTTGGCGGAAGCGTGTAAAATC	TCCCTAGTCCCGCGCAATGAGC	60
CHK1	TCATCCATTTCTAACAAATTCACTT	TGGGCTATCAATGGAAGAAAA	60
SOX2	TGCTGCCTCTTTAAGACTAGGG	TCGGGCTCCAAACTTCTCT	60
BMI-1	CTGGTTGCCCATTGACAGC	CAGAAAATGAATGCGAGCCA	60
β-CATENIN	GAAACGGCTTTCAGTTGAGC	TTCCATCATGGGGTCCATAC	60
KLF-4	TACCAAGAGCTCATGCCACC	CGCGTAATCACAAGTGTGG G	60
OCT-4	GCTGGAGCAAAACCCGGAGG	TCGGCCTGTGTATATCCCAGGGTG	60
NANOG	TCTCCAACATCCTGAACCTCAGCT	GAGGCCTTCTGCGTCACACCA	60
LIN28	AGCATGCAGAAGCGCAGATCAA	GCTACCATATGGCTGATGCTCT	60
LIN28B	GCCCCTTGGATATTCCAGTC	TGACTCAAGGCCTTTGGAAG	60
ALDH1A1	CCACTCACTGAATCATGCCA	TGAGCCAGTCACCTGTGTTC	60
E-CADHERIN	AGCCCCGCCTTATGATTCTCTG	TGCCCCATTCGTTCAAGTAGTCAT	60
VIMENTIN	AATGACCGCTTCGCCAAC	CCGCATCTCCTCCTCGTAG	60
ABCG2	TGGTGTTTCCTTGTGACACTG	TGAGCCTTTGGTTAAGACC G	60

β-ACTIN	TACATGGCTGGGGTGTTGAA	AAGAGAGGCATCCTCACCCT	60
ATR	GGAGGAGTTTTGGCCTCCACA	CTGCGAGGCACTAGTCAACC	60
EED	CTGTAGGAAGCAACAGAGTTACC	CATAGGTCCATGCACAAGTGT	60
SUZ12	GCATTGCCCTTGGTGTACTC	TGGTCCGTTGCGACTAAAA	60

Table S5 The primers used for CHIP.

Primer	Upstream	Downstream
CHK1 CHIP primer-1#	ACCAAGCCCGACTAATTCCT	CTCAGGGATAGGCAGGATCA
CHK1 CHIP primer-2#	GCATTGTTTTGGAGCTGGTT	TGAGAGCCGACTGTGAAGAA
CHK1 CHIP primer-3#	GAGTCCCAGCCCTTCCTTTC	GGAGACAAATGCTTTCCGGC
CHK1 CHIP primer-4#	GAATTGAGCAATTGGGAGGA	TGAAATTCTGCCCTCCTCAG
CHK1 CHIP primer-5#	CTTGGGAGTGGCGATTGTGA	GAGCACCTCGGCTGTAAAAG
CHK1 CHIP primer-6#	GCCTTCGTTTTCTGAGTGCA	AACTTCGTGTCCCTTCCAGC
HOXA2 CHIP primer	TGGGAGTGTGTGTGTGTGTG	TAATGACTGCAGGCGTCAGA

Table S6 Patient characteristics and association with EZH2 or pCHK1 expression.

Patient/tumor characteristics	Total N (%)	EZH2 expression N (%)		P-*value*	χ^2	pCHK1 expression N (%)		P-*value*	χ^2
		High	Low			High	Low		
Age at diagnosis (y）				1.00	0.08			1.00	0.01
<45	8 (100)	4 (50)	4 (50)			3 (37.5)	5 (62.5)		
≥45	36 (100)	16 (44.44)	20 (55.56)			13 (36.11)	23 (63.89)		
FIGO stage				0.013	6.56			0.067	3.89
Ⅰ and Ⅱ	10 (100)	1 (10)	9 (90)			1 (10)	9 (90)		
Ⅲ and Ⅳ	34 (100)	19 (55.89)	15 (39.47)			15 (44.12)	19 (55.88)		
Histology				0.436	1.15			0.224	2.41

Serous	36 (100)	15 (41.67)	21 (58.33)			15 (41.67)	21 (58.33)		
Others	8 (100)	5 (62.5)	3 (37.5)			1 (12.5)	7 (87.5)		
Tumor categories				0.484	0.49			0.278	2.10
I	11 (100)	6 (54.55)	5 (45.45)			2 (18.18)	9 (81.82)		
II	33 (100)	14 (42.42)	19 (57.58)			14 (42.42)	19 (57.58)		
Chemotherapy resistance				0.001	10.95			0.001	13.44
Yes	15 (100)	12 (80)	3 (20)			11 (73.33)	4 (26.67)		
No	29 (100)	8 (27.59)	21 (72.41)			5 (17.24)	24 (82.76)		
Relapse				0.02	5.37			0.007	7.24
Yes	27 (100)	16 (59.26)	11 (40.74)			14 (51.85)	13 (48.15)		
No	17 (100)	4 (23.53)	13 (76.47)			2 (11.76)	15 (88.24)		
Death				0.017	5.65			0.000	12.99
Yes	20 (100)	13 (65)	7 (35)			13 (65)	7 (35)		
No	24 (100)	7 (29.17)	17 (70.83)			3 (12.5)	21 (87.5)		

Table S7 Incidence of tumors by SK-3rd cells and SKOV3 cells in node mice.

Number of Cells Inoculated		10^4	10^5
SK-3rd cells	Untransduced	6/6	6/6
	shNC	6/6	6/6
	shEZH2	0/6	0/6
SKOV3 cells		0/6	3/6

Table S8 Logistic univariate analysis for chemo-resistance.

Variables	P
Age at diagnosis	0.55
FIGO stage	0.1
Histology	0.55

Tumor categories	0.58
EZH2 expression	0.002
pCHK1 expression	0.00

Table S9 Logistic multivariate analysis for chemo-resistance.

Variables	P	Exp(B)
pCHK1 expression	0.005	2.31
EZH2 expression	0.017	2.05

Table S10 Cox univariate analysis for relapse.

Variables	P
Age at diagnosis	0.51
FIGO stage	0.05
Histology	0.38
Tumor categories	0.38
Chemo-resistance	0.07
EZH2 expression	0.04
pCHK1 expression	0.00

Table S11 Cox multivariate analysis for relapse.

Variables	P	Hazard ratio
pCHK1 expression	0.002	4.26
EZH2 expression	0.409	1.42
FIGO	0.225	2.19
